# Natural Acceptor of Coumarin‐Isomerized Red‐Emissive BioAIEgen for Monitoring Cu^2+^ Concentration in Live Cells via FLIM

**DOI:** 10.1002/advs.202307078

**Published:** 2023-12-15

**Authors:** Xu‐Min Cai, Shouji Li, Wen‐Jin Wang, Yuting Lin, Weiren Zhong, Yalan Yang, Fritz E. Kühn, Ying Li, Zheng Zhao, Ben Zhong Tang

**Affiliations:** ^1^ Jiangsu Co‐Innovation Center of Efficient Processing and Utilization of Forest Resources International Innovation Center for Forest Chemicals and Materials College of Chemical Engineering Nanjing Forestry University Nanjing 210037 P.R.China; ^2^ Clinical Translational Research Center of Aggregation‐Induced Emission The Second Affiliated Hospital School of Medicine School of Science and Engineering Shenzhen Institute of Aggregate Science and Technology The Chinese University of Hong Kong, Shenzhen (CUHK‐Shenzhen) Guangdong 518172 P.R.China; ^3^ Molecular Catalysis Department of Chemistry & Catalysis Research Center School of Natural Sciences Technische Universität München D‐85747 München Germany; ^4^ Innovation Research Center for AIE Pharmaceutical Biology School of Pharmaceutical Sciences and the Fifth Affiliated Hospital Guangzhou Medical University Guangzhou 511436 P.R.China

**Keywords:** BioAIEgen, copper sensor, coumarin, FLIM, red emission

## Abstract

Artificial aggregation‐induced emission luminogens (AIEgens) have flourished in bio‐applications with the development of synthetic chemistry, which however are plagued by issues like singularity in structures and non‐renewability. The unique structures and renewability of biomass moieties can compensate for these drawbacks, but their properties are hard to design and regulate due to their confined structures. Therefore, it appears to be a reasonable approach to derive AIEgens from abundant biomass (BioAIEgens), integrating the bilateral advantages of both synthetic and natural AIEgens. In this work, the blue‐violet emissive coumarin with its lactone structure serving as a rare natural acceptor, is utilized to construct donor‐π‐acceptor typed BioAIE isomers incorporating the propeller‐like and electron‐donating triphenylamine (TPA) unit. The results show that **Cm‐*p*‐TPA** undergoes charge transfer with its keto form, emitting red light at 600 nm, which can be applied to monitor Cu^2+^ concentration during mitophagy using fluorescence lifetime imaging microscopy because of the excellent biocompatibility, photostability, and specific recognition to Cu^2+^. This work not only demonstrates the feasibility of utilizing positional isomerization to modulate excited‐state evolutions and resultant optical properties, but also provides evidence for the rationality of constructing biologically‐active BioAIEgens via a biomass‐derivatization concept.

## Graphical abstract

This work not only demonstrates the feasibility of utilizing positional isomerization to modulate excited‐state evolutions and produce a red‐emissive BioAIEgen to be applied to monitor the Cu^2+^ concentration in live cells using FLIM, but also provides evidence for the rationality of constructing biologically‐active BioAIEgens via a biomass‐derivatization concept.

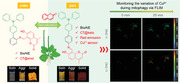

## 1 Introduction

**Figure 1:**
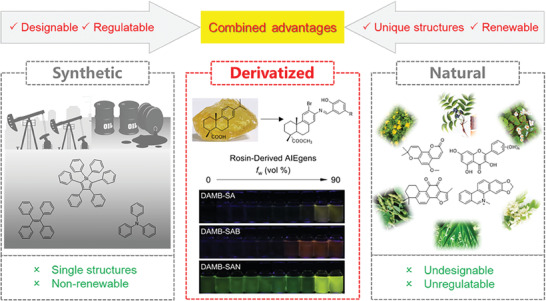
The comprehensive advantages of BioAIEgens derived from biomass.

Currently, petrochemical‐based aggregation‐induced emission (AIE) materials with outstanding optical properties are widely applied in phototherapy, as bio‐sensors, in anti‐counterfeiting, and several other fields.^[^ ^1^ ^]^ However, these synthetic AIE materials are mostly derived from molecular scaffolds such as tetraphenylethylene (TPE) and triphenylamine (TPA), which have limitations with respect to accessible structures and are non‐renewable. This may hamper the further development of novel AIE materials. AIE materials derived from biomass or possessing natural skeletons (BioAIEgens)^[^ ^2^ ^]^ in contrast can compensate for these limitations, due to their unique natural structures, their renewability, and their inherent biocompatibility.^[^ ^3^ ^]^ In recent years, more and more natural products, such as quercetin,^[^ ^4^ ^]^ kaempferol,^[^ ^5^ ^]^ berberine,^[^ ^6^ ^]^ tanshinone,^[^ ^7^ ^]^ and coumarin,^[^ ^8^ ^]^ have been found to exhibit AIE properties, showing great potential in bioimaging and therapeutics. However, most of the purely natural BioAIEgens are difficult to extract and confined to fixed structures, making it difficult to optimize their luminescence properties. Therefore, the utilization of proper and abundant biomass moieties as starting materials for tunable, multifunctional, and renewable BioAIEgens with novel structures through chemical modification is of great interest (**Figure** **1**).^[^ ^2^, ^9^ ^]^

AIEgens with long‐wavelength emission are commonly used in the fields of bioimaging and therapeutics due to their advantages such as deep tissue penetration, low photo‐damage, and low scattering effects.^[^ ^10^ ^]^ BioAIEgens always show good bio‐compatibility, making them excellent candidates for vivo‐applications. However, as far as we know, only Tang's group reported a purely natural case of tanshinone‐based BioAIEgen with red emission for photodynamic therapy.^[^ ^7^ ^]^ Therefore, more red‐emissive BioAIEgens via molecular design strategies for bio‐applications based on biomass resources would be highly desirable.

**Scheme 1:**
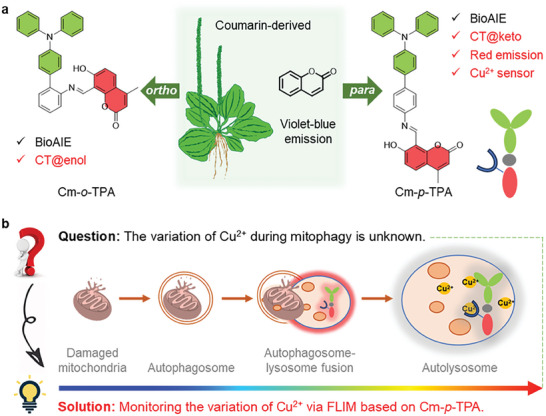
a) Molecular design of coumarin‐derived BioAIEgens. CT@enol, and CT@keto represent for CT in the enol and keto forms, respectively. b) Diagram of monitoring the concentration variation of Cu^2+^ during mitophagy via FLIM based on Cm‐*p*‐TPA in cells. FLIM is especially adapted to the turn‐off sensors compared to turn‐on ones.

It is well known that long‐wavelength emission can be achieved by constructing a donor‐π‐acceptor (D‐π‐A) structure to induce a charge transfer (CT) effect.^[^ ^11^ ^]^ Coumarin is a common natural product with an unsatisfying blue‐violet emission^[^ ^2c^ ^]^ that cannot be utilized for practical applications. Nevertheless, its lactone structure can be regarded as a rare natural acceptor^[^ ^12^ ^]^ for constructing D‐π‐A typed red‐emissive BioAIEgens. Besides, the subtle differences in structural isomers may result in the distinction of CT properties. For instance, Wang et al. have studied the effect of structural isomerization on the resultant near‐infrared properties, and found that the *para*‐substituted isomer possesses stronger D–A interaction and red‐shifted emission.^[^ ^13^ ^]^ In addition, the influence of *ortho*, *meta*, and *para* positions on the intramolecular CT effect of phenothiazine‐fused benzothiazoles has been investigated, discovering that the *para*‐substituted isomer exhibits stronger intramolecular CT characteristics due to a smaller torsion angle and enhanced conjugation.^[^ ^14^ ^]^ Therefore, we assumed it is possible to achieve red‐emissive coumarin‐based BioAlEgens as specific metal sensors with chelating salicylaldehyde Schiff base structures via the introduction of TPA at variable positions of the π bridge with tunable CT properties (**Scheme** **1**a).

Copper is an essential trace element in biological systems and plays specific biological roles in organisms.^[^ ^15^ ^]^ The imbalance of copper inside cells can disrupt cellular functions, hence the copper content is strictly regulated. Previous studies have shown that copper ions play a significant role in mitochondrial biosynthesis and the respiratory chain.^[^ ^16^ ^]^ Mitophagy is an intercellular self‐renewal process that selectively eliminates redundant or damaged mitochondria, which indeed can lead to changes in copper ion concentration and potentially affect the entire copper ion transfer network. However, to the best of our knowledge, there have been no reports on the changes of copper ion concentration during mitophagy. Traditional intensity‐based probes are limited by their concentration‐dependent characteristics and the extent of dye uptaken by cells. Especially with respect to turn‐off sensors, they can neither be applied in intensity‐based probing methods nor provide direct visual observation. In contrast, the fluorescence lifetime imaging microscopy (FLIM) method has attracted widespread attention because of its high‐resolution and sensitivity to the cellular microenvironment, independent of the concentration of the dye. However, the reported Cu^+^ sensor is completely synthetic and not economically applicable.^[^ ^17^ ^]^ Thus, by using the red‐emissive and biocompatible BioAIEgen as a specific copper sensor, in conjunction with the FLIM method, it should be possible to detect and visualize the variation of copper during mitophagy and disclose the role of copper in the cellular mitophagy (Scheme 1b).

In this work, the coumarin‐based position‐isomerized BioAIEgens (**Cm‐*o*‐TPA** and **Cm‐*p*‐TPA**) were successfully obtained through introducing a propeller‐shaped and electron‐donating TPA group on the *ortho*‐ and *para*‐position of the coumarin structure. The results demonstrate that the coumarin structure can indeed work as a natural acceptor to construct D‐π‐A molecules and that the positional isomerism shows a significant influence on the CT effect. The *ortho*‐substituted **Cm‐*o*‐TPA** exhibits CT in the enol state, while the *para*‐substituted **Cm‐*p*‐TPA** exhibits CT in the keto state, with the latter resulting in red emission. Furthermore, based on the biocompatibility, red emission, and specific recognition toward Cu^2+^ of **Cm‐*p*‐TPA**, the FLIM method can be successfully employed to monitor the concentration changes of Cu^2+^ during mitophagy, indicating a continuous increase in Cu^2+^ concentration during mitophagy.

## 2 Results and Discussion

### 2.1 Synthesis and Characterization

As shown in Schemes S1 and S2 (Supporting Information), **Cm‐CHO** was reacted with **TPA‐*o*‐NH_2_**, **TPA‐*p*‐NH_2_**, **Ph‐NH_2_**, **Ph‐*o*‐NH_2_**, and **Ph‐*p*‐NH_2_** through a simple Schiff base reaction to obtain the target molecules **Cm‐*o*‐TPA** and **Cm‐*p*‐TPA**, as well as the control molecules **Cm‐Ph**, **Cm‐*o*‐Ph**, and **Cm‐*p*‐Ph**. All compounds were characterized using ^1^H NMR, ^13^C NMR, and HRMS (Figures S1–S16, Supporting Information).

### 2.2 ESIPT‐CT‐AIE Properties of Cm‐*o*‐TPA and Cm‐*p*‐TPA

**Figure 2:**
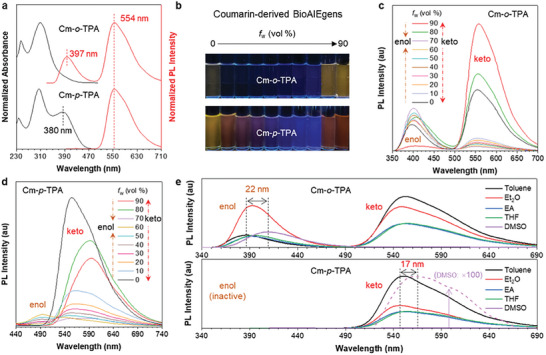
a) Normalized absorption and PL spectra of Cm‐*o*‐TPA and Cm‐*p*‐TPA in pure THF solution. Concentration: 10 µm. Cm‐*o*‐TPA (*λ*_ex_: 309 nm) and Cm‐*p*‐TPA (*λ*_ex_: 380 nm). b) Fluorescence photographs of Cm‐*o*‐TPA and Cm‐*p*‐TPA in tetrahydrofuran (THF)/H_2_O mixtures with different water fractions (*f*_w_) taken under 365 nm UV irradiation. Concentration: 10 µm. c,d) PL spectra of Cm‐*o*‐TPA c) (*λ*_ex_: 309 nm) and Cm‐*p*‐TPA d) (*λ*_ex_: 380 nm) in THF/H_2_O mixtures with different *f*_w_. Concentration: 10 µm. e) PL spectra of Cm‐*o*‐TPA and Cm‐*p*‐TPA in solvents with different polarities. Concentration: 10 µm. The absorption maximum of each solution was chosen as its excitation wavelength.

The photophysical properties of **Cm‐*o*‐TPA** and **Cm‐*p*‐TPA** in their molecular state have been explored at first. In **Figure** **2**a, **Cm‐*p*‐TPA** exhibits an additional absorption peak at 380 nm compared to **Cm‐*o*‐TPA**, possibly attributable to the CT absorption peak.^[^ ^18^ ^]^ In the photoluminescence (PL) spectra, the two emission peaks at 397 and 554 nm for **Cm‐*o*‐TPA** can be assigned to the enol and keto peaks, respectively, typically accompanied with the excited‐state intramolecular proton transfer (ESIPT) process.^[^ ^19^ ^]^ **Cm‐*p*‐TPA** only exhibits a single keto peak,^[^ ^20^ ^]^ indicating that **Cm‐*p*‐TPA** is more favorable for ESIPT. It is assumed that the *para*‐substituted **Cm‐*p*‐TPA** with less steric hindrance is more inclined to experience ESIPT and CT processes at the molecular level. As shown in Table S1 (Supporting Information), the quantum yields (QYs) of **Cm‐*o*‐TPA** and **Cm‐*p*‐TPA** in tetrahydrofuran (THF)/H_2_O mixtures with different water fractions (*f*_w_) = 0% are 3.10% and 5.72%, respectively, which are of no big difference. However, when visually inspecting the fluorescence pictures, **Cm‐*o*‐TPA** shows weaker emission than **Cm‐*p*‐TPA**. That might because **Cm‐*o*‐TPA** exhibits both enol and keto emissions, with the enol emission falling in the UV‐violet region not sensitive to the naked eye. **Cm‐*p*‐TPA** exhibits a sole keto emission in the visible region, hence exhibiting stronger emission to the naked eye. Next, the photophysical properties of two isomers in their aggregated states have been further investigated. As shown in Figure 2b, it can be clearly observed that the fluorescence intensity of both isomers in THF/H_2_O mixtures exhibits a trend of decreasing and then increasing with increased *f*_w_, indicating their CT and AIE characteristics.^[^ ^21^ ^]^ **Cm‐*p*‐TPA** exhibits a more pronounced redshift in its aggregated state, which may be attributed to the different CT evolution processes. To further verify this, the corresponding PL test was carried out (Figure 2c,d). With the increase of *f*_w_, the intensities of the enol peaks of both isomers exhibit an initial enhancement followed by a decrease, while the keto peaks show the opposite trend (Figure S17, Supporting Information). This is consistent with the fluorescence phenomenon, indicating that aggregation at high *f*_w_ (Figure S18, Supporting Information) facilitates the ESIPT process and promotes the formation of keto structures, leading to the ESIPT‐induced AIE performance.^[^ ^9^, ^20^ ^]^ The concentration effect further confirms their AIE properties (Figure S19, Supporting Information). Interestingly, the enol peaks of **Cm‐*o*‐TPA** exhibit a more pronounced wavelength shift with increased *f*_w_ compared to the keto peaks, while the opposite way is observed for **Cm‐*p*‐TPA**. This indicates that the positional isomerism may have a significant impact on the excited‐state CT process. Through a solvent effect test (Figure 2e; Figure S20, Supporting Information), it is found that with changes in polarity, the emission wavelength of the enol peaks of **Cm‐*o*‐TPA** differ by 22 nm, while those of the keto peaks of **Cm‐*p*‐TPA** show a significant shift of 17 nm. Therefore, **Cm‐*o*‐TPA** indeed exhibits CT in the enol form, while **Cm‐*p*‐TPA** shows CT in the keto form. The above‐described results suggest that a *para*‐substitution is more inclined to experience ESIPT to result in the keto structure, subsequently followed by the CT process. The synergistic excited‐state evolutions of ESIPT and CT can result in the red emission of **Cm‐*p*‐TPA**. This conclusion is further supported by a study of control molecules. **Cm‐Ph** without donor moiety only exhibits ESIPT‐AIE performance (Figure S21, Supporting Information), while the donor incorporation of a benzene ring in both **Cm‐*o*‐Ph** and **Cm‐*p*‐Ph** results in similar spectroscopic properties to those of **Cm‐*o*‐TPA** and **Cm‐*p*‐TPA** (Figures S22 and S23, Supporting Information), further confirming that the *para*‐substitution strategy on the D‐π‐A scaffold is more conducive to initiate CT process after ESIPT, hence leading to the red emission.

### 2.3 Photophysical Properties in the Aggregate States

**Figure 3:**
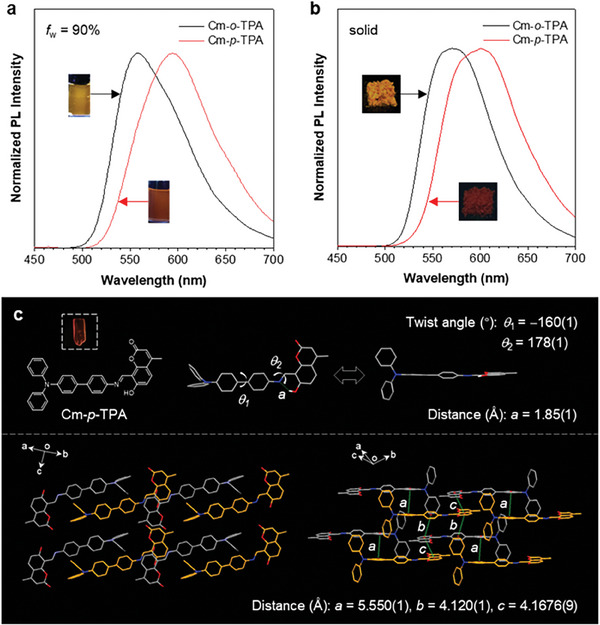
a,b) Normalized PL spectra of Cm‐*o*‐TPA (*λ*_ex_: 360 nm) and Cm‐*p*‐TPA (*λ*_ex_: 380 nm) in THF/H_2_O mixtures with *f*_w_ = 90% a) and as solid b). Inset: fluorescence photographs of Cm‐*o*‐TPA and Cm‐*p*‐TPA at respective states, taken under 365 nm UV irradiation. c) Molecular conformation (top) and packing (bottom) of Cm‐*p*‐TPA. Inset: a red single crystal of Cm‐*p*‐TPA.

Based on the variable CT processes revealed in the molecular state, the photophysical properties in the aggregate states have been further studied. In the water rich fraction (*f*_w_ = 90%), **Cm‐*p*‐TPA** exhibits an emission peak at ≈600 nm, exhibiting redder emission compared to **Cm‐*o*‐TPA** (wavelength: 550 nm) (**Figure** **3**a). Interestingly, both **Cm‐*o*‐TPA** (wavelength: 570 nm) and **Cm‐*p*‐TPA** (wavelength: 600 nm) exhibit similar fluorescence properties (Figure 3b) in the crystalline states verified by the strong and sharp diffraction peaks found in the powder X‐ray (PXRD) diffraction measurement (Figure S24, Supporting Information). When comparing their emission intensity at both molecular and aggregate states (Table S1, Supporting Information), **Cm‐*o*‐TPA** shows stronger emission in the aggregate states (QY = 3.10%, 9.52%, and 12.70% at *f_w_* = 0%, 90%, and solid state, respectively), most probably due to the restriction of molecular motion. Interestingly, **Cm‐*p*‐TPA** exhibits the opposite way, with the QY value larger at molecular level (5.72%) than that in the aggregate states (1.09% and 0.60% at *f_w_* = 90% and solid state, respectively). This might be attributed from its planar conformation at aggregate state that can contribute to the charge transfer in the keto emission, hence causing a redshift and more energy dissipation through non‐radiative channels, resulting in a decreased QY. A single crystal of **Cm‐*p*‐TPA** was used for further clarification (Figure 3c; Table S2, Supporting Information). According to the conformation of **Cm‐*p*‐TPA**, the intramolecular hydrogen bond length in the crystal structure is 1.85(1) Å, favoring the occurrence of an ESIPT process.^[^ ^20^ ^]^ The torsion angles (−160(1)° and 178(1)°) indicate planar conformation, which facilitates the CT process to cause red emission. Moreover, different views of molecular packing reveal a staggered arrangement and larger intermolecular distances (5.550(1), 4.120(1), and 4.1676(9) Å), suggesting weak intermolecular interactions that account for the weak fluorescence. Nevertheless, the crystallographic results reaffirm that the red emission of **Cm‐*p*‐TPA** is caused by the CT process after ESIPT via a positional substitution strategy.

### 2.4 Spectroscopic and Lifetime Studies of Cm‐*p*‐TPA to Cu^2+^ In Vitro

**Figure 4:**
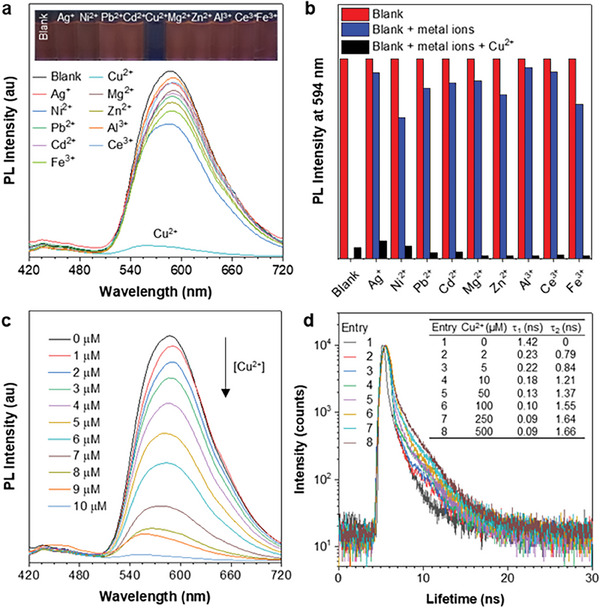
a) The PL spectra of Cm‐*p*‐TPA (10 µm) in the presence of different metal ions (100 µm) including Cu^2+^ in THF/PBS (v/v = 20/80, pH 7.4). Inset: the photography of the corresponding metal ion mixture solutions under a 365 nm UV lamp. b) PL intensities of Cm‐*p*‐TPA (10 µm) alone (blank), blank + various metal ions (100 µm), and blank + metal ions + Cu^2+^ (100 µm) in THF/PBS (v/v = 20/80, pH 7.4) solution, *λ*_ex_ = 380 nm. c) Fluorescence titration spectra of Cm‐*p*‐TPA (10 µm) upon addition of a Cu^2+^ (0–10 µm) in THF/PBS (v/v = 20/80, pH 7.4) solution. d) The fluorescence lifetime decay curves of Cm‐*p*‐TPA (10 µm) with increasing amounts of Cu^2+^ in PBS buffer (pH 7.4). Inset: fitting parameters.

The above‐described BioAIEgens featuring ─OH and ─C═N─ groups can coordinate with metal ions to form stable hexacoordinated chelates, resulting in a potential metal ion sensor.^[^ ^22^ ^]^ Due to the smaller steric hindrance of **Cm‐*p*‐TPA** compared to **Cm‐*o*‐TPA**, it may exhibit stronger coordinating ability with metal ions. Hence, **Cm‐*p*‐TPA** has been selected as a sensor for the in vitro studies. According to the results shown in **Figure** **4**a and Figure S25 (Supporting Information), it can be observed that both the absorption and PL spectra of **Cm‐*p*‐TPA** have been affected by Cu^2+^ when ten different metal ions (Ag^+^, Ni^+^, Pb^2+^, Cd^2+^, Cu^2+^, Mg^2+^, Zn^2+^, Al^3+^, Ce^2+^, and Fe^3+^) are added to the THF/PBS (*f*_w_ = 80%) mixture, suggesting the specific recognition of Cu^2+^ with **Cm‐*p*‐TPA**. The fluorescence quenching of **Cm‐*p*‐TPA** upon complexation with Cu^2+^ may be attributed to ligand‐metal charge transfer.^[^ ^23^ ^]^ Comparative experiments further confirm that the *para*‐position exhibits stronger selectivity toward Cu^2+^ (vide supra). As shown in the fluorescence images (Figure S26c,d, Supporting Information), it is evident that the fluorescence of **Cm‐*p*‐TPA** is quenched after adding Cu^2+^, while **Cm‐*o*‐TPA** doesn't show such behavior, indicating the stronger coordination ability of **Cm‐*p*‐TPA**. In addition, both the absorption and PL spectra of **Cm‐*o*‐TPA** (Figure S26a,c, Supporting Information) remain almost unchanged after adding Cu^2+^, whereas those of **Cm‐*p*‐TPA** (Figure S26b,d, Supporting Information) undergo noticeable changes, further indicating the stronger coordination of **Cm‐*p*‐TPA** with Cu^2+^. Additionally, Figure 4b demonstrates that even in the presence of other metal ions, the addition of Cu^2+^ significantly reduces the fluorescence intensity of **Cm‐*p*‐TPA**, suggesting that the recognition of Cu^2+^ by **Cm‐*p*‐TPA** is not influenced by other metal ions. Furthermore, it can be observed that the PL intensity of **Cm‐*p*‐TPA** gradually decreases as the concentration of Cu^2+^ increases from 0 to 10 µm (Figure 4c). The calculated lower limit of detection of **Cm‐*p*‐TPA** is 0.205 × 10^−6^ m (S/N = 3) (Figure S27, Supporting Information), suitable as Cu^2+^ sensor in biological systems.^[^ ^24^ ^]^ Since **Cm‐*p*‐TPA** demonstrates fluorescence quenching upon complexation with Cu^2+^, it is more adaptable to monitor the changes of Cu^2+^ concentration using the FLIM method. The lifetime changes of **Cm‐*p*‐TPA** upon binding with Cu^2+^ in vitro have been first tested (Figure 4d; Figure S28, Supporting Information). As shown in Figure 4d, when 0 to 500 µm Cu^2+^ was added to **Cm‐*p*‐TPA**, the fluorescence lifetime of **Cm‐*p*‐TPA** significantly increased after binding with Cu^2+^. Two lifetime components (*τ*_1_ and *τ*_2_) were extracted from the decay fitting values. The short lifetime *τ*_1_ can be assigned to **Cm‐*p*‐TPA**, while the long lifetime *τ*_2_ is assigned to the complex formed by **Cm‐*p*‐TPA** and Cu^2+^. The decrease in *τ*_1_ indicates gradual coordination between **Cm‐*p*‐TPA** and Cu^2+^, resulting in fluorescence quenching and a shorter lifetime. The increase in *τ*_2_ indicates the increasing amount of the complex formed by **Cm‐*p*‐TPA** and Cu^2+^, leading to fluorescence quenching and a longer lifetime. This indicates that the FLIM method can be applied to monitor changes of Cu^2+^ concentration in vitro.

### 2.5 Targeted Imaging of Lysosomes by Cm‐*p*‐TPA Nanoparticles

Considering **Cm‐*p*‐TPA** has potential in Cu^2+^ sensing in vitro, it is a good choice to utilize this natural scaffold‐derived BioAIEgen for monitoring in biological systems. To clarify the utilization form of **Cm‐*p*‐TPA**, the long‐term chemical and optical stability of **Cm‐*p*‐TPA** (with/without Pluronic *F127* encapsulation) in Dulbecco's Modified Eagle Medium (DMEM) with 10% fetal bovine serum (FBS) as a supplement have been examined. As shown in Figure S29 (Supporting Information), we first examined the chemical stability of **Cm‐*p*‐TPA** within 48 h and observed a gradual decrease in absorption intensity over time, suggesting that its chemical stability was suboptimal. However, upon encapsulating it with *F127*, the chemical stability was significantly improved within these 48 h. Then we evaluated the photostability and observed a decreasing trend in both the absorption and fluorescence intensities of **Cm‐*p*‐TPA** with increasing light exposure time (Figure S30a,c,d,f, Supporting Information). Nevertheless, when combined with *F127*, the absorption intensity remained almost unchanged, and the fluorescence intensity only showed a slight decrease (Figure S30b,c,e,f, Supporting Information). These findings highlight the advantages of incorporating *F127* to enhance the chemical and photostability of **Cm‐*p*‐TPA**, thus the Pluronic *F127* encapsulated **Cm‐*p*‐TPA** nanoparticles (NPs) verified by dynamic light scattering (DLS) and transmission electron microscope (TEM) results (Figure S31, Supporting Information) have been applied for all intracellular experiments since either **Cm‐*p*‐TPA** itself or **Cm‐*p*‐TPA** NPs exhibits a response to Cu^2+^ in a fluorescence quenching effect (Figure S32, Supporting Information). First, both the cytotoxicity and photocytotoxicity of **Cm‐*p*‐TPA** NPs have been investigated by MTT assays (Figures S33 and S34, Supporting Information). The results confirm that the IC50 values for non‐light and light treatment groups are found to be above 200 and 180 µm, respectively, demonstrating their superior biocompatibility to be suitably applied in cell imaging. In addition, its photostability in HeLa cells is tested (Figure S35, Supporting Information). With increasing irradiation time from 0 to 1200 s, the fluorescence intensity keeps nearly invariable, suggesting that **Cm‐*p*‐TPA** NPs exhibit good photostability. Furthermore, we have evaluated the targeting ability of **Cm‐*p*‐TPA** NPs to cellular organelles in HeLa cells (Figure S36, Supporting Information). Through the co‐localization experiments of **Cm‐*p*‐TPA** NPs with LysoTracker Deep Red FM (LTDR) and MitoTracker Green FM (MTG), it is found that **Cm‐*p*‐TPA** NPs could target lysosomes with a good Pearson correlation coefficient (PCC: 0.84). The above described cell experimental results indicate that **Cm‐*p*‐TPA** NPs have low cytotoxicity, photocytotoxicity, and good photostability in HeLa cells, and they can image lysosomes their in.

### 2.6 FLIM Imaging of Cu^2+^ Changes During Mitophagy Using Cm‐*p*‐TPA Nanoparticles

**Figure 5:**
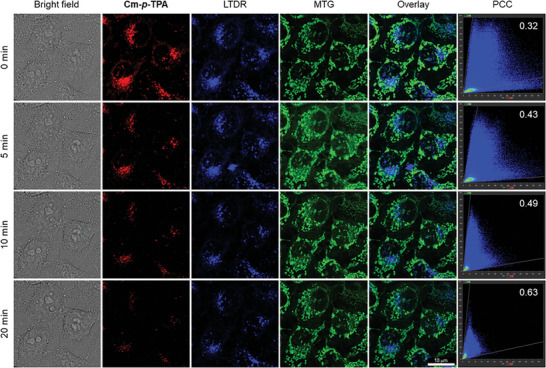
Monitoring the mitophagy process using Cm‐*p*‐TPA NPs (10 µm). The HeLa cells were incubated with Cm‐*p*‐TPA NPs (10 µm) for 12 h before starved in D‐Hanks for 2 h. Then, the cells were incubated with LTDR (200 nm) and MTG (200 nm) for 15 min before imaging using confocal microscopy. Cm‐*p*‐TPA NPs: *λ*_ex_ = 405 nm; *λ*_em_ = 590 ± 20 nm. MTG: *λ*_ex_ = 490 nm; *λ*_em_ = 516 ± 20 nm. LTDR: *λ*_ex_ = 633 nm; and *λ*_em_ = 670 ± 20 nm. Scale bars: 10 µm.

Currently, copper ions play an important role in mitochondrial biosynthesis and respiratory chain function. Mitophagy may lead to changes in copper ion concentration and may affect the entire copper ion transfer network. Since **Cm‐*p*‐TPA** containing natural coumarin structure, red emission, and recognition of Cu^2+^ can image lysosome specifically, it has the potential to monitor copper ion changes during mitophagy. To ensure that mitochondrial autophagy is successfully initiated, the expression level of key signaling proteins for mitophagy was determined by western blot assays (Figure S37a, Supporting Information) and the morphological changes of mitochondria for mitophagy was monitored by TEM (Figure S37b, Supporting Information). The results of western blot indicate that nutritional deprivation for 2 h could induce significant up‐regulation of mitophagy associated Parkin and PINK1. Furthermore, up‐regulation of LC3‐II indicates the formation of autophagosome, which also could be verified by the autophagic structure in the TEM images. Considering the co‐existence of Cu^+^ in biological systems, we have conducted a control experiment (Figure S38, Supporting Information) to investigate whether **Cm‐*p*‐TPA** is also affected by Cu^+^. The results show that the fluorescence of **Cm‐*p*‐TPA** aggregates is not quenched when Cu^+^ is added, indicating that it is not affected by Cu^+^. Next, **Cm‐*p*‐TPA** NPs have been applied to the actual mitophagy process in HeLa cells. **Cm‐*p*‐TPA** NPs were co‐incubated with HeLa cells for 12 h. Then, the HeLa cells were starved in a D‐Hanks buffer for 2 h to induce apoptosis and trigger mitophagy. The cells were labeled with LTDR, and MTG 15 min before imaging by confocal microscopy. In **Figure** **5**, the Overlay and PCC columns of LTDR and MTG demonstrate that at 0 min, the PCC between mitochondria and lysosomes in HeLa cells is low (0.32), indicating that the mitophagy process has just started. After 20 min, the PCC between mitochondria and lysosomes increases to 0.63, indicating that the mitophagy process is more than halfway complete. Within the 0 to 20 min timeframe, it is observed that the fluorescence intensity locating in the lysosomes gradually decreases. These results suggest the release of Cu^2+^ during the mitophagy process since both the chemical‐ and photo‐bleaching effects are out of consideration due to pronounced chemical‐ and photo‐stability (vide supra). It is inferred that Cu^2+^ coordinates with **Cm‐*p*‐TPA**, and the concentration of Cu^2+^ gradually increases during mitophagy.

**Figure 6:**
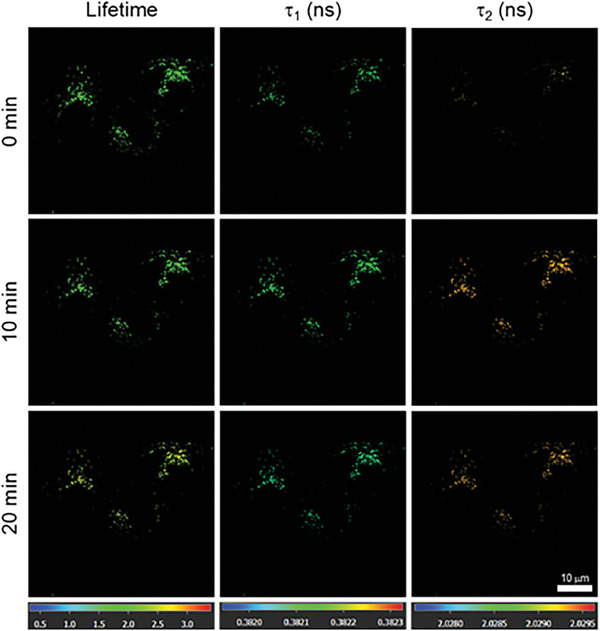
The lifetime changes of Cm‐*p*‐TPA NPs (10 µm) during the starvation‐inducing mitophagy process of HeLa cells. Scale bar: 10 µm.

To shed more light, FLIM has been used to monitor the changes in Cu^2+^ concentration during mitophagy in HeLa cells. Changes in the fluorescence lifetime of **Cm‐*p*‐TPA** NPs within the lysosomes (**Figure** **6**) also exhibits similar results to the in vitro experiments. Within 0 to 20 min, the lysosomes stained with **Cm‐*p*‐TPA** NPs show a gradual decrease in the short lifetime *τ*_1_, resulting in a color change from yellow‐green to green in the imaging. On the other hand, the long lifetime *τ*_2_ gradually increases, resulting in a color change from light orange to dark orange in the imaging. As a result, it is clear that the concentration of Cu^2+^ increases gradually during the mitophagy process in HeLa cells, in addition to the fluorescence quenching in lysosome imaging (vide supra).

## 3 Conclusion

In summary, we successfully transformed the natural acceptor of blue‐violet emissive coumarin into its derived BioAIEgens (**Cm‐*o*‐TPA** and **Cm‐*p*‐TPA**) with distinct ESIPT‐CT‐AIE properties by introducing TPA through positional isomerization. Specifically, **Cm‐*o*‐TPA** exhibits CT in its enol form, while **Cm‐*p*‐TPA** exhibits CT in the keto form, leading to red emission. Additionally, single‐crystal analysis shows that the introduction of *para*‐substituted TPA results in a planar conformation for stronger CT, leading to a redshift. Furthermore, based on the good biocompatibility, red emission, and specific recognition of Cu^2+^ by **Cm‐*p*‐TPA** NPs, it has been successfully applied to monitor the changes in Cu^2+^ concentration during the mitophagy process in HeLa cells using the FLIM method.

## Conflict of Interest

The authors declare no conflict of interest.

## Supporting information

Supporting Information

## Data Availability

The data that support the findings of this study are available from the corresponding author upon reasonable request.
