# Supplementary material for: Natural Acceptor of Coumarin‐Isomerized Red‐Emissive BioAIEgen for Monitoring Cu2+ Concentration in Live Cells via FLIM
Source: Adv Sci (Weinh). 2023 Dec 15;11(9):2307078. doi: 10.1002/advs.202307078 (PMC10916553; doi:10.1002/advs.202307078)
Supplement: Supplementary file 1 — Supporting Information [file ADVS-11-2307078-s001.pdf]

ADVANCED SCIENCE
Open Access

Supporting Information

for *Adv. Sci.*, DOI 10.1002/advs.202307078

Natural Acceptor of Coumarin-Isomerized Red-Emissive BioAIEgen for Monitoring Cu^{2+} Concentration in Live Cells via FLIM

Xu-Min Cai, Shouji Li, Wen-Jin Wang, Yuting Lin, Weiren Zhong, Yalan Yang, Fritz E. Kühn, Ying Li, Zheng Zhao* and Ben Zhong Tang**

Supporting Information

Natural Acceptor of Coumarin-Isomerized Red-Emissive BioAIEgen for Monitoring Cu^{2+} Concentration in Live Cells via FLIM

Xu-Min Cai, Shouji Li, Wen-Jin Wang, Yuting Lin, Weiren Zhong, Yalan Yang, Fritz E. Kühn, Ying Li, Zheng Zhao,* Ben Zhong Tang**

Table of Contents

Materials and Instrumentations 4

Synthesis and characterization 5

Preparation of samples for AIE measurements 7

Preparation of metal ion detection experiments 7

Preparation of Cm-p-TPA nanoparticles (Cm-p-TPA NPs) 8

Cell culturing 8

Cell imaging 8

Cytotoxicity assay 9

Scheme S1. The synthetic routes of Cm-o-TPA and Cm-p-TPA. 10

Scheme S2. Synthetic procedures of Cm-Ph, Cm-o-Ph, and Cm-p-Ph. All reactions are performed in MeOH at 95 °C for 3 h. 11

Figure S1. ^{1}H NMR spectrum of Cm-CHO. 11

Figure S2. ^{1}H NMR spectrum of Cm-o-TPA. 12

Figure S3. ^{13}C NMR spectrum of Cm-o-TPA. 12

Figure S4. HR-MS spectrum of Cm-o-TPA. 13

Figure S5. ^{1}H NMR spectrum of Cm-p-TPA. 13

Figure S6. ^{13}C NMR spectrum of Cm-p-TPA. 14

Figure S7. HR-MS spectrum of Cm-p-TPA. 14

Figure S8. ^{1}H NMR spectrum of Cm-Ph. 15

Figure S9. ^{13}C NMR spectrum of Cm-Ph. 15

Figure S10. HR-MS spectrum of Cm-Ph. 16

Figure S11. ^{1}H NMR spectrum of Cm-o-Ph. 16

Figure S12. ^{13}C NMR spectrum of Cm-o-Ph. 17

Figure S13. HR-MS spectrum of Cm-o-Ph. 17

Figure S14. ^{1}H NMR spectrum of Cm-p-Ph. 18

Figure S15. ^{13}C NMR spectrum of Cm-p-Ph. 18

Figure S16. HR-MS spectrum of Cm-p-Ph. 19

Table S1. The QY data of Cm-*o*-TPA (λ_{ex} : 360 nm) and Cm-*p*-TPA (λ_{ex} : 380 nm) in THF/H_2O mixtures with f_w = 0%, 90%, and as solid. Inset: fluorescence photographs of Cm-*o*-TPA and Cm-*p*-TPA in THF/H_2O mixtures with f_w = 0%, 90%, and as solid taken under 365 nm UV irradiation. 19

Table S2. Crystallographic data for compound Cm-*p*-TPA. 20

Figure S17. (a and c) The enol (α_{AIE}) emission plots (versus f_w) of Cm-*o*-TPA (a) and Cm-*p*-TPA (c). $\alpha_{AIE} = I/I_0$, I_0 = PL intensity in pure THF. (b and d) The plots of the keto (α_{AIE}) emission plots (versus f_w) of Cm-*o*-TPA (b) and Cm-*p*-TPA (d). $\alpha_{AIE} = I/I_0$, I_0 = PL intensity in pure THF. 21

Figure S18. DLS results of Cm-*o*-TPA (a) and Cm-*p*-TPA (b) in THF/H_2O mixtures with f_w = 90% (10 μM). 21

Figure S19. (a and c) PL spectra of Cm-*o*-TPA (a) and Cm-*p*-TPA (c) in THF solution with different concentrations. Cm-*o*-TPA (λ_{ex}: 360 nm) and Cm-*p*-TPA (λ_{ex}: 380 nm). (b and d) The plots of the emission intensity at the maximum versus the concentration of Cm-*o*-TPA (b) and Cm-*p*-TPA (d) in THF, I_0 = PL intensity in THF solution (1 μM). 22

Figure S20. (a and b) Absorption spectra of Cm-*o*-TPA (a) and Cm-*p*-TPA (b) in solvents with different polarities. Concentration: 10 μM. (c) Normalized enol-emission PL spectra of Cm-*o*-TPA in solvents with different polarities. (d) Normalized keto-emission PL spectra of Cm-*o*-TPA and Cm-*p*-TPA in solvents with different polarities. 23

Figure S21. (a) Absorption and PL spectra of Cm-Ph in dilute ACN solution (10 μM). (λ_{ex}: 365 nm). (b) PL spectra of Cm-Ph in ACN/H_2O mixtures with different water fractions (f_w). Concentration: 10 μM. (λ_{ex}: 365 nm). Inset: The fluorescence photographs of Cm-Ph in ACN/H_2O mixtures with different water fractions (f_w). (c) The enol, keto (α_{AIE}) emission plots (versus f_w) of Cm-Ph. $\alpha_{AIE} = I/I_0$, I_0 = PL intensity in pure ACN. (d) PL spectra of Cm-Ph as solid taken under 365 nm UV light. Inset: Fluorescence photographs Cm-Ph in solid states taken under 365 nm UV irradiation. 24

Figure S22. (a and b) Normalized absorption (a) and PL (b) spectra of Cm-*o*-Ph and Cm-*p*-Ph in pure THF solution. Concentration: 10 μM. Cm-*o*-Ph (λ_{ex}: 317 nm) and Cm-*p*-Ph (λ_{ex}: 337 nm). Insert: chemical structure of Cm-*o*-Ph and Cm-*p*-Ph. 24

Figure S23. (a and b) PL spectra of (a) Cm-*o*-Ph and (b) Cm-*p*-Ph in solvents with different polarities. Concentration: 10 μM. The absorption maximum of each solution was chosen as its excitation wavelength. 25

Figure S24. PXRD spectra of Cm-*o*-TPA and Cm-*p*-TPA. 25

Figure S25. The absorption spectra of Cm-*p*-TPA (10 μM) in the presence of different metal ions (100 μM) including Cu^{2+} in THF/PBS (v/v = 20/80, pH = 7.4). 25

Figure S26. (a and b) The absorption spectra before and after adding Cu^{2+} (100 μM) to Cm-*o*-TPA (10 μM) (a) and Cm-*p*-TPA (10 μM) (b) in THF/PBS (v/v = 20/80, pH = 7.4) solution. Inset: the photography before and after adding Cu^{2+} to Cm-*o*-TPA and Cm-*p*-TPA in THF/PBS (v/v = 20/80, pH = 7.4) solution under day light. (c and d) The PL spectra before and after adding Cu^{2+} (100 μM) to Cm-*o*-TPA (10 μM) (c) and Cm-*p*-TPA (10 μM) (d) in THF/PBS (v/v = 20/80, pH =

7.4) solution, Cm-*o*-TPA, λ_{ex} = 360 nm; Cm-*p*-TPA, λ_{ex} = 380 nm. I_0 = PL intensity before adding Cu^{2+}. I = PL intensity after adding Cu^{2+}. Inset: the photography before and after adding Cu^{2+} to Cm-*o*-TPA and Cm-*p*-TPA in THF/PBS (v/v = 20/80, pH = 7.4) solution under 365 nm UV lamp. 26

Figure S27. The linear relationships between the fluorescence intensity of Cm-*p*-TPA at 594 nm and Cu^{2+} concentration. 27

Figure S28. The lifetime decay curves of Cm-*p*-TPA with increasing amount of Cu^{2+} in PBS buffer (pH = 7.4). 27

Figure S29. (a and b) Absorption spectra of Cm-*p*-TPA (a) and Cm-*p*-TPA NPs (b) in biological medium including FBS and DMEM for 48 h. (c) The plots of the absorption intensity at the maximum versus time. A_0 = Absorption intensity at 0 h. Concentration: 10 μM. 27

Figure S30. Absorption and PL spectra of Cm-*p*-TPA (a and d) and Cm-*p*-TPA NPs (b and e) in biological medium including FBS and DMEM under white light exposure for a duration of 30 minutes. (c and f) The plots of the absorption intensity (c) and PL intensity (f) at the maximum versus time under white light exposure. A_0 = Absorption intensity at 0 min. I_0 = PL intensity at 0 min. Concentration: 10 μM. 28

Figure S31. Hydrodynamic diameter of the Cm-*p*-TPA NPs and their distribution measured by DLS. The insert is the TEM image of the Cm-*p*-TPA NPs. Scale bar: 500 nm. 28

Figure S32. PL spectra before and after adding Cu^{2+} (100 μM) to Cm-*p*-TPA (10 μM) (a) and Pluronic *F127* encapsulated Cm-*p*-TPA (b) in biological medium including FBS and DMEM. 29

Figure S33. (a and b) Cell viabilities of HeLa (a) and LO2 (b) cells after treatment with indicated concentration of Cm-*p*-TPA NPs for 24 h. The control groups were treated with the same volume of PBS buffer without Cm-*p*-TPA NPs. 29

Figure S34. (a and b) Cell viabilities of HeLa (a) and LO2 (b) cells after treatment with indicated concentration of Cm-*p*-TPA NPs for 24 h before irradiated using a 405 nm led array (10 mW cm^{-2}, 10 min). The control groups were treated with the same volume of PBS buffer without Cm-*p*-TPA NPs. 29

Figure S35. Photostability of Cm-*p*-TPA NPs (10 μM) in HeLa cells under continuous one-photon laser irradiation. Cm-*p*-TPA: λ_{ex} = 405 nm; λ_{em} = 590 ± 20 nm. 30

Figure S36. Co-Localization of Cm-*p*-TPA NPs (10 μM, 2 h) in HeLa cells coincubated with LysoTracker® Deep Red FM (LTDR, 200 nM, 15 min) and MitoTracker® Green FM (MTG, 200 nM, 15min). Cm-*p*-TPA NPs: λ_{ex} = 405 nm; λ_{em} = 590 ± 20 nm. LTDR: λ_{ex} = 633 nm; λ_{em} = 670 ± 20 nm. MTG: λ_{ex} = 490 nm; λ_{em} = 516 ± 20 nm. Scale bars: 20.0 μm. 30

Figure S37. (a) Western blot analysis of starvation inducing mitophagy. PINK1, PTEN induced putative kinase 1. LC3, microtubule-associated protein 1 light chain 3. (b) Representative TEM images showing the ultrastructure of HeLa cells mitophagy brings after incubating in D-Hank buffer for 2 h. The cells in control groups are incubated in DMEM with 10% FBS as supplements. The red rectangle indicates the enlarged region. M represents for mitophagy.. 31

Figure S38. The photography of Cm-*p*-TPA (10 μM) before and after adding Cu^{+} in THF/H_2O (v/v = 20/80) under 365 nm UV lamp. 31

References. 32

Author Contributions. 32

Materials and Instrumentations

Materials

Cm-CHO is derived from 7-Hydroxy-4-methylcoumarin (Energy Chemical, 98%) according to literature methods.[1] 2-Bromoaniline (Aladdin, 97%), 4-Bromoaniline (Aladdin, 97%), 2-Aminodiphenyl (Energy Chemical, 97%), 4-Aminobiphenyl (Energy Chemical, 99%), 4-(Diphenylamino) phenylboronic acid (Aladdin, 98%), and Tetrakis (triphenylphosphine) palladium (Energy Chemical, 99%) were used without purification. Chloroform-d_3 ($CDCl_3$, CIL, 99.8%), and all other organic solvents are purchased and used without further purification unless otherwise claimed. Fetal bovine serum (FBS), Trypsin, Dulbecco's modified eagle medium (DMEM) and Penicillin-Streptomycin Solution (PS) were purchased from Hyclone Laboratoreis Inc, USA. Phosphate buffered saline (PBS) and dimethyl sulfoxide (DMSO) were purchased from Sigma Aldrich, USA. 3-(4,5-dimethyl-2-thiazolyl)-2,5-diphenyl-2-H-tetrazolium bromide (MTT) was brought from Sigma Aldrich. LysoTracker Deep Red (LTDR) and MitoTracker Green (MTG) were obtained from ThermoFisher Scientific, USA. Antibodies for LC3 (ab192890), PINK1 (ab216144), Parkin (ab77924) and β-actin (ab8226) were obtained from Abcam. For bioimaging experiments, the tested compounds were dissolved in DMSO as stock solution and diluted into the expected experiment concentration containing 1% (v/v) DMSO as needed. In each experiment, vehicle control (1% DMSO) was used as the reference group. Milli-Q water was from a Milli-Q purification system (Merck Millipore, Germany).

Instrumentations

NMR measurements were performed on a Bruker AVANCE-III-600 spectrometer (^1H, 600 MHz; ^{13}C, 150 MHz) with $CDCl_3$ as solvent. Melting points were measured on an OptiMelt MPA100 apparatus (SRS, USA) without correction. High resolution mass spectra (HRMS) were on a Q Exactive mass spectrometer (Thermo Scientific) operating in an ESI mode. The UV–visible absorption spectra of all the compounds were collected with a Shimadzu UV2450 spectrometer. The absorption spectra of Cm-*p*-TPA and Cm-*p*-TPA NPs in biological medium including FBS and DMEM for 48 h were collected with a Lambda 365 UV-Vis Spectrophotometer. The absorption spectra of Cm-*p*-TPA and Cm-*p*-TPA NPs in biological medium including FBS and DMEM under white light exposure for a duration of 30 minutes were collected with a Shimadzu UV-3600 Plus UV-Vis-NIR spectrophotometer. Photoluminescence spectra were recorded on a Fluoromax-4 spectrofluorometer. The absolute

fluorescence quantum yields (QY) were determined on a Fluoromax-4 spectrometer by a Quanta-ϕ integrating sphere. Powder X-ray diffraction (PXRD) experiments were tested on a Rigaku Ultima IV diffractometer with Cu Kα radiation. Dynamic light scattering (DLS) measurements were performed on a Malvern Zetasizer Nano ZS analyzer. Single crystal data of Cm-*p*-TPA were collected on a Xcalibur, Eos, Gemini diffractometer. Cu Kα radiation (λ = 1.54184). Bioimaging experiments were conducted using the Leica STELLARIS 5 & STELLARIS 8 confocal microscopy. MTT assays were recorded on FlexStation 3 Multi-Mode Microplate Reader.

Synthesis and characterization

Synthesis of Cm-*o*-TPA

2-Bromoaniline (149 mg, 0.865 mmol), 4-(Diphenylamino) phenylboronic acid (250 mg, 0.865 mmol), K_2CO_3 (1 mL, 2 mmol), and Tetrakis (triphenylphosphine) palladium (33 mg, 0.03 mmol) are slowly added to a solution of toluene (2 mL). All feeding processes are carried out under N_2 atmosphere. After 6 h of stirring at 115 °C, the reaction mixture is extracted with DCM and purified by silica gel column chromatography (eluents are petroleum ether/ethyl acetate, volume ratio: 20/1) to obtain the brownish intermediate TPA-*o*-NH_2 (yield: 70%). Then, TPA-*o*-NH_2 (148 mg, 0.440 mmol) is slowly added to a vigorously stirred solution of Cm-CHO (75 mg, 0.367 mmol) in 25 mL of methanol. The reaction mixture is refluxed for 3 h and cooled to room temperature afterwards. Cm-*o*-TPA is precipitated as yellow solid. The product can be obtained by filtration, and the yield is 80%. Melting point (m. p.): 229.7-230.1 °C. ^{1}H NMR (600 MHz, $CDCl_3$-d_3): δ 14.42 (s, 1H), 9.39 (s, 1H), 7.56 (d, J = 8.9 Hz, 1H), 7.47-7.37 (m, 4H), 7.28 (s, 1H), 7.27 (s, 2H), 7.25 (s, 2H), 7.24 (s, 1H), 7.18 (d, J = 7.6 Hz, 4H), 7.14 (d, J = 8.5 Hz, 2H), 7.02 (t, J = 7.3 Hz, 2H), 6.85 (d, J = 8.9 Hz, 1H), 6.14 (s, 1H), 2.41 (s, 3H). ^{13}C NMR (150 MHz, $CDCl_3$-d_3): δ 166.17, 156.25, 153.32, 147.82, 144.86, 137.24, 133.16, 130.59, 130.54, 129.32, 129.11, 128.56, 127.83, 124.50, 123.63, 122.93, 118.76, 114.84, 111.16, 111.03, 107.29, 19.08. HRMS (ESI) calcd. for $C_{35}H_{26}N_2O_3$ $[M + H]^+$: 523.1943, found: 523.1996.

Synthesis of Cm-*p*-TPA

4-Bromoaniline (149 mg, 0.865 mmol), 4-(Diphenylamino) phenylboronic acid (250 mg, 0.865 mmol), K_2CO_3 (1 mL, 2 mmol), and Tetrakis (triphenylphosphine) palladium (33 mg, 0.03 mmol) are slowly added to a solution of toluene (2 mL). All feeding processes are

carried out under N_2 atmosphere. After 6 h of stirring at 115 °C, the reaction mixture is extracted with DCM and purified by silica gel column chromatography (eluents are petroleum ether/ethyl acetate, volume ratio: 20/1) to obtain the brownish intermediate TPA-*p*-NH_2 (yield: 60%). Then, TPA-*p*-NH_2 (148 mg, 0.440 mmol) is slowly added to a vigorously stirred solution of Cm-CHO (75 mg, 0.367 mmol) in 25 mL of methanol. The reaction mixture is refluxed for 3 h and cooled to room temperature afterwards. Cm-*p*-TPA is precipitated as red solid. The product can be obtained by filtration, and the yield is 75%. Red single crystals suitable for X-ray crystallographic measurements have been obtained by slow evaporation from the mixed solution of DCM/Methanol (v/v, 1/1). Melting point (m. p.): 261.3-262.0 °C. ^{1}H NMR (600 MHz, $CDCl_3$-*d₃*): δ 15.24 (s, 1H), 9.41 (s, 1H), 7.66 (d, J = 8.4 Hz, 2H), 7.58 (d, J = 9.0 Hz, 1H), 7.51 (d, J = 8.5 Hz, 2H), 7.45 (d, J = 8.4 Hz, 2H), 7.30 (s, 1H), 7.28 (s, 2H), 7.27 (s, 1H), 7.16 (s, 3H), 7.14 (d, J = 1.7 Hz, 3H), 7.05 (t, J = 7.3 Hz, 2H), 6.93 (d, J = 9.0 Hz, 1H), 6.15 (s, 1H), 2.42 (s, 3H). ^{13}C NMR (150 MHz, $CDCl_3$-d_3) δ 155.71, 147.68, 129.45, 127.76, 127.72, 124.69, 123.84, 123.24, 121.88, 115.19, 111.06, 19.11. HRMS (ESI) calcd. for $C_{35}H_{26}N_2O_3$ $[M + H]^+$: 523.1943, found: 523.1998.

[CCDC 2252826 contains the supplementary crystallographic data for this paper. These data can be obtained free of charge from The Cambridge Crystallographic Data Centre via www.ccdc.cam.ac.uk/data_request/cif.]

Synthesis of Cm-Ph

53.3 μL (0.59 mmol) aniline and 100 mg (0.49 mmol) Cm-CHO are dissolved in 25 mL of methanol. The reaction mixture is refluxed for 3 h and cooled to room temperature afterwards. Cm-Ph is precipitated as orange solid. The product can be obtained by filtration, and the yield is 87%. Melting point (m. p.): 161.4-161.9 °C. ^{1}H NMR (600 MHz, $CDCl_3$-d_3): δ 15.12 (s, 1H), 9.34 (s, 1H), 7.57 (d, J = 9.0 Hz, 1H), 7.45 (t, J = 7.8 Hz, 2H), 7.37 (d, J = 7.5 Hz, 2H), 7.32 (t, J = 7.3 Hz, 1H), 6.90 (d, J = 9.0 Hz, 1H), 6.12 (s, 1H), 2.41 (s, 3H). ^{13}C NMR (150 MHz, $CDCl_3$-d_3): δ 1166.96, 160.33, 156.57, 129.69, 129.41, 127.83, 121.41, 115.15, 111.06, 19.05. HRMS (ESI) calcd for $C_{17}H_{13}NO_3$ $[M + H]^+$: 280.0895, found: 280.0957.

Synthesis of Cm-*o*-Ph

2-Aminodiphenyl (49.8 mg, 0.294mmol) and (50mg, 0.245mmol) Cm-CHO are dissolved in 15mL of methanol. The reaction mixture is refluxed for 3h and cooled to room temperature

afterwards. Cm-*o*-Ph is precipitated as orange solid. The product can be obtained by filtration, and the yield is 42.5%. ^{1}H NMR (600 MHz, $CDCl_3$-d_3): δ 14.30 (s, 1H), 9.39 (s, 1H), 7.52 (d, J = 9.0 Hz, 1H), 7.43 (tt, J = 17.0, 8.1 Hz, 9H), 6.80 (d, J = 8.9 Hz, 1H), 6.13 (s, 1H), 2.39 (s, 3H). ^{13}C NMR (150 MHz, $CDCl_3$-d_3): δ 165.98, 160.38, 156.61, 153.32, 144.86, 137.44, 130.92, 129.77, 129.15, 128.88, 128.38, 127.86, 127.62, 118.85, 114.84, 111.09, 19.06. HRMS (ESI) calcd for $C_{23}H_{17}NO_3$ $[M + H]^+$: 356.1208, found: 356.1267.

Synthesis of Cm-*p*-Ph

4-Aminobiphenyl (49.8mg, 0.294mmol) and 50mg (0.245mmol) Cm-CHO are dissolved in 15mL of methanol. The reaction mixture is refluxed for 3h and cooled to room temperature afterwards. Cm-*o*-Ph is precipitated as red solid. The product can be obtained by filtration, and the yield is 58.6%. ^{1}H NMR (600 MHz, $CDCl_3$-d_3): δ 15.17 (s, 1H), 9.40 (s, 1H), 7.68 (d, J = 8.4 Hz, 2H), 7.62 (d, J = 7.3 Hz, 2H), 7.58 (d, J = 9.0 Hz, 1H), 7.47 (dd, J = 7.9, 4.5 Hz, 4H), 7.37 (t, J = 7.4 Hz, 1H), 6.92 (d, J = 8.9 Hz, 1H), 6.14 (s, 1H), 2.41 (s, 3H). ^{13}C NMR (150 MHz, $CDCl_3$-d_3): δ 166.91, 156.12, 145.58, 129.42, 129.03, 128.32, 127.77, 127.14, 121.89, 115.15, 111.10, 107.09, 19.10. HRMS (ESI) calcd for $C_{23}H_{17}NO_3$ $[M + H]^+$: 356.1208, found: 356.1270.

Preparation of samples for AIE measurements

Cm-*o*-TPA and Cm-*p*-TPA: The tetrahydrofuran (THF)-water mixture with different water fractions (f_w, the volume percentage of water in THF/H_2O mixtures) were prepared at room temperature and the concentration was maintained at 1.0×10^{-5} M. The PL measurement of the mixture was performed immediately.

Cm-Ph: The acetonitrile (ACN)-water mixture with different water fractions (f_w, the volume percentage of water in ACN/H_2O mixtures) were prepared at room temperature and the concentration was maintained at 1.0×10^{-5} M. The PL measurement of the mixture was performed immediately.

Preparation of metal ion detection experiments

A stock solution of Cm-*p*-TPA at a concentration of 1.0×10^{-5} M was prepared in PBS = 80% (PBS, the volume percentage of PBS buffer (pH = 7.40) in THF/PBS mixtures) for UV-vis

and fluorescence measurements. Other analytical solutions were prepared in deionized water. The interferential solutes included some common metal ions (Ag^+, Ni^+, Pb^{2+}, Cd^{2+}, Cu^{2+}, Mg^{2+}, Zn^{2+}, Al^{3+}, Ce^{2+}, and Fe^{3+}) as nitrate salts.

Preparation of Cm-*p*-TPA nanoparticles (Cm-*p*-TPA NPs)

Cm-*p*-TPA (1 mg) and the surfactant Pluronic *F-127* (3 mg) were dissolved in THF, respectively, and prepared as a mixed solution, which was then gently added into DI water (9 mL). The mixtures were sonicated in an ice bath for 2 min and then magnetically stirred for 12 h and dialyzed for 48 h in DI water. Finally, the resulting nanoparticles suspension was concentrated to 10 mg mL^{-1} and disinfected by adopting a PES membrane with 0.22 μm filter pore size (Merck Millipore, Ireland) for subsequent experimentation.

Cell culturing

HeLa cell were cultured in DMEM or RPMI1640 medium with 10% FBS, 100 μg mL^{-1} streptomycin and 100 U mL^{-1} penicillin as supplement. Cells were cultured at 37 °C in a humidified incubator with an atmosphere of 5% CO_2 and 95% air (normoxia, 5% carbon dioxide.

Cell imaging

Subcellular Localization

HeLa cells were seeded in culture dishes (Corning) for 24 h, and then incubated with Cm-*p*-TPA NPs (10 μM). The cells were then stained with MTG (100 nM) or LTDR (200 nM) for 15 min. The cells were washed with PBS twice and imaged by confocal microscopy immediately. λ_{ex} = 405 nm (Cm-*p*-TPA NPs); 490 nm (MTG); 633 nm (LTDR); λ_{em} = 590 ± 20 nm (Cm-*p*-TPA NPs); 510 ± 20 nm (MTG); 700 ± 20 nm (LTDR).

Western Blotting

HeLa cells were seeded in 6-well plates and incubated for 24 hours. Subsequently, the cells were treated with D-Hanks buffer for different time intervals (0, 0.5, 1.0, 1.5, 2.0, and 2.5 hours). After that, the cells were harvested and lysed to extract proteins. The protein concentration was determined using BCA assays (bicinchoninic acid). Equal amounts of total

cellular proteins (30 μg) were separated on SDS-PAGE and transferred onto polyvinylidene difluoride membranes (Millipore, MA, USA). The membranes were then blocked and incubated overnight at 4 °C with primary antibodies followed by appropriate horseradish peroxidase-conjugated secondary antibody incubation. Images were captured and analyzed using a JP-K600 imaging station (China).

Transmission electron microscopy (TEM) measurement

HeLa cells were seeded in 10 cm culture dishes for 24 h, and then replaced the culture medium with the D-Hanks buffer for 2 h. Subsequently, cells were trypsinized and washed with cold PBS three times, and harvested by centrifuging at 1000 rcf for 5 min. After that, cells were fixed with glutaric dialdehyde. The samples were stained with Osmium tetroxide before imaging. All the images were obtained by the Eversmart Jazz program (Scitex).

Mitophagy Tracking

HeLa cells were seeded in culture dishes (Corning) for 24 h and incubated the with Cm-*p*-TPA NPs (10 μM) for another12 h before replaced the culture medium with the D-Hanks buffer for 2 hours to induce the mitophagy of cells. Then the cells were labeled with MTG (100 nM) and LTDR (200 nM) for 15 min. After that the cells were washed with PBS and D-Hanks bufferwas replenished before imaging by confocal microscopy. λ_{ex} = 405 nm (Cm-*p*-TPA NPs); 490 nm (MTG); 633 nm (LTDR); λ_{em} = 590 ± 20 nm (Cm-*p*-TPA NPs); 510 ± 20 nm (MTG); 700 ± 20 nm (LTDR).

Photostability of Cm-*p*-TPA NPs

HeLa cells were seeded in culture dishes (Corning) for 24 h, and then incubated with Cm-*p*-TPA NPs (10 μM) for 12 h, after which imaging was performed every 30 s for 8 min. λ_{ex} = 405 nm (Cm-*p*-TPA NPs); λ_{em} = 590 ± 20 nm (Cm-*p*-TPA NPs).

Cytotoxicity assay

The cytotoxicity of the Cm-*p*-TPA NPs was measured by the MTT assay. In detail, HeLa cells or LO2 were seeded in 96-well plates, and incubated for 24 h before being treated with the tested compounds for 20 h. Then, 20 μL MTT (5 mg mL^{-1}) was added to each well, and incubated for another 4 h. The medium was replaced with 150 DMSO (per each well). The

absorbance at 595 nm was determined using a microplate reader. The percentage of cell viability was calculated using the equation: (mean OD of treated cells/mean OD of control cells) × 100%.

For photocytoxicity: HeLa cells or LO2 were seeded in 96-well plates for 24 h. Subsequently, the cells treated with various concentrations of Cm-*p*-TPA NPs for 12 h, followed by irradiated with a 405 nm led arrays (10 mW, 10 min). After an additional incubation period of 8 hours, each well was supplemented with 20 μL MTT solution (5 mg mL^{-1}) and further incubated for another 4 hours. Finally, the toxicity was measured as mentioned above.

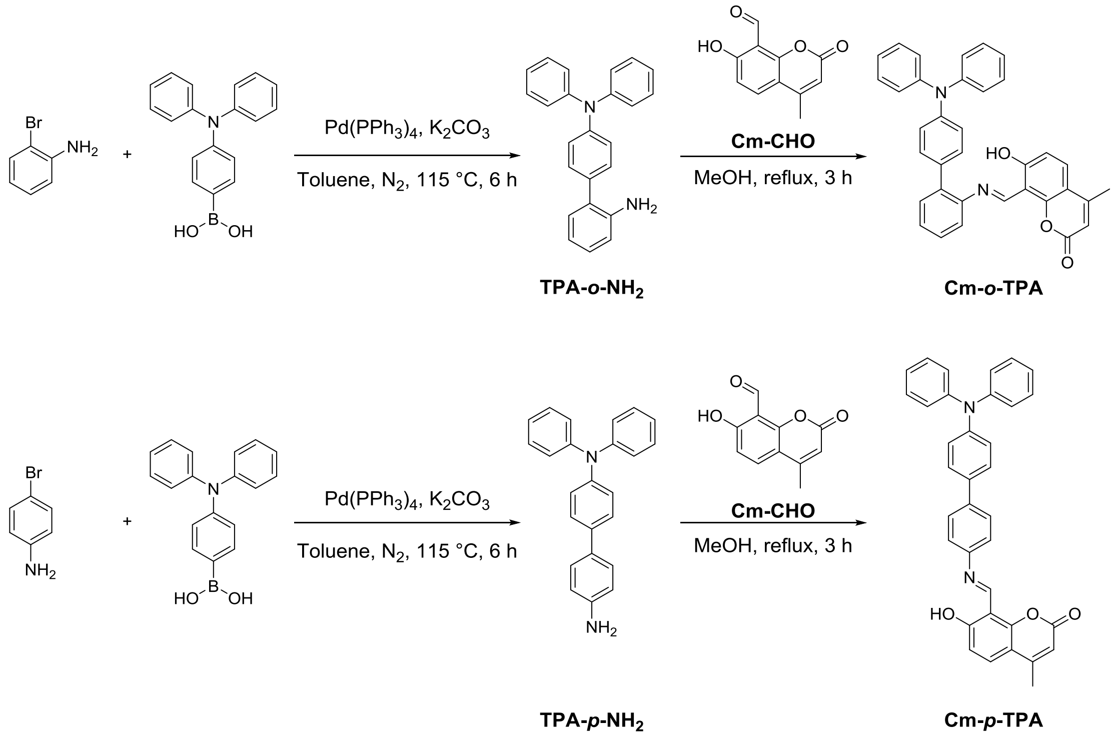

Scheme S1. The synthetic routes of Cm-*o*-TPA and Cm-*p*-TPA.

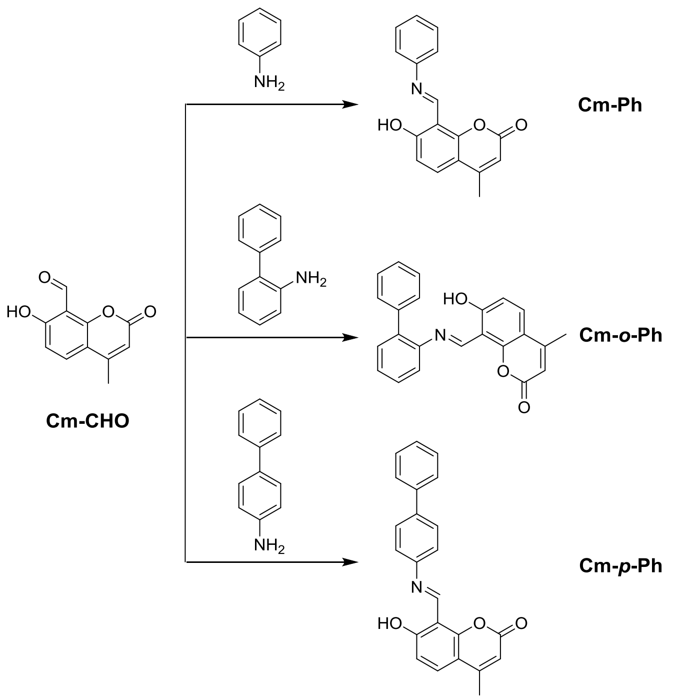

Scheme S2. Synthetic procedures of Cm-Ph, Cm-*o*-Ph, and Cm-*p*-Ph. All reactions are performed in MeOH at 95 °C for 3 h.

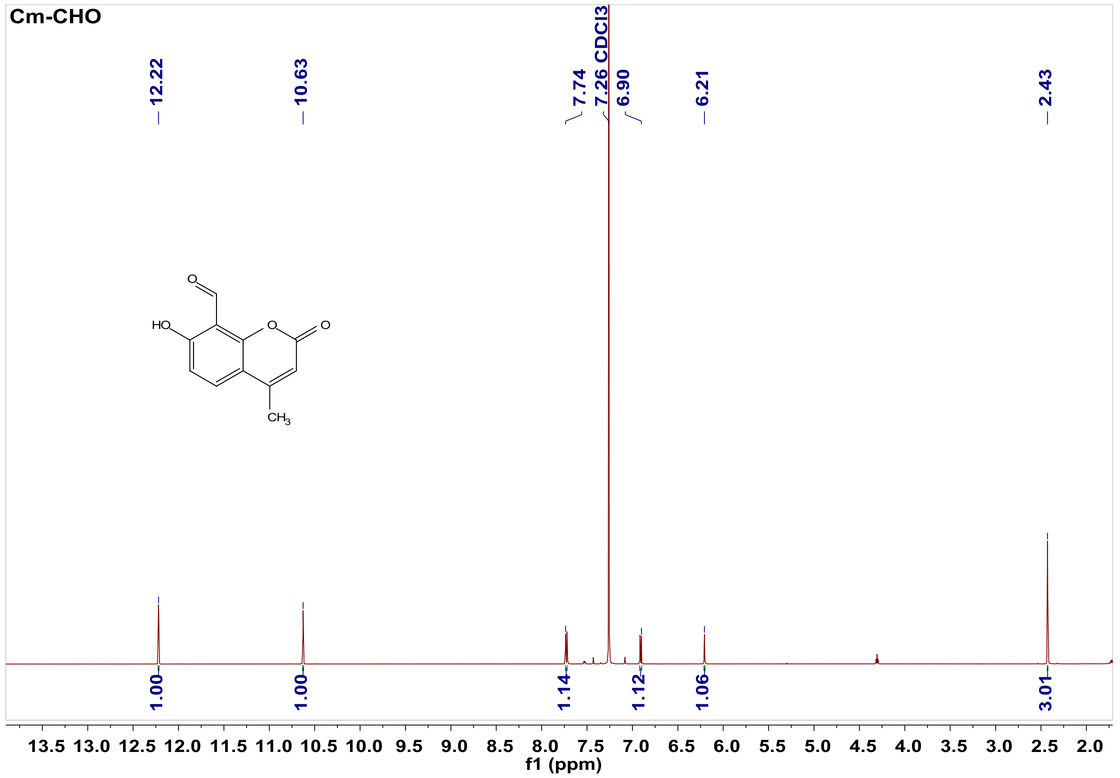

Figure S1. ^{1}H NMR spectrum of Cm-CHO.

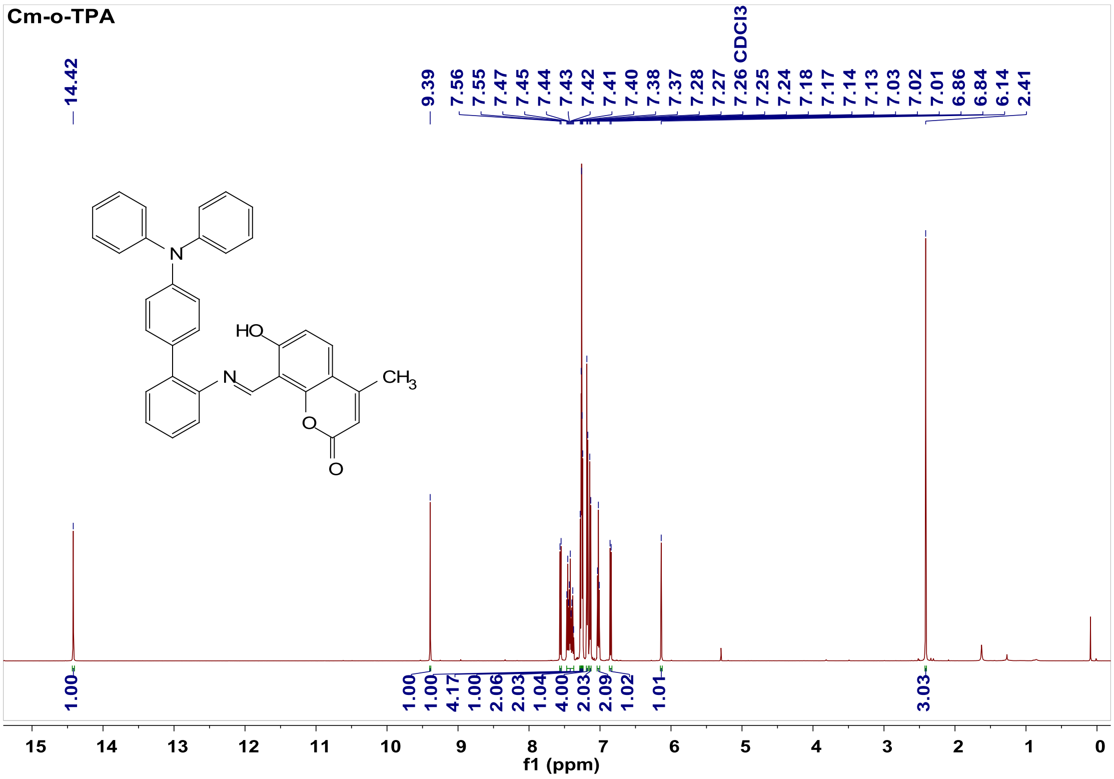

Figure S2. ^{1}H NMR spectrum of Cm-*o*-TPA.

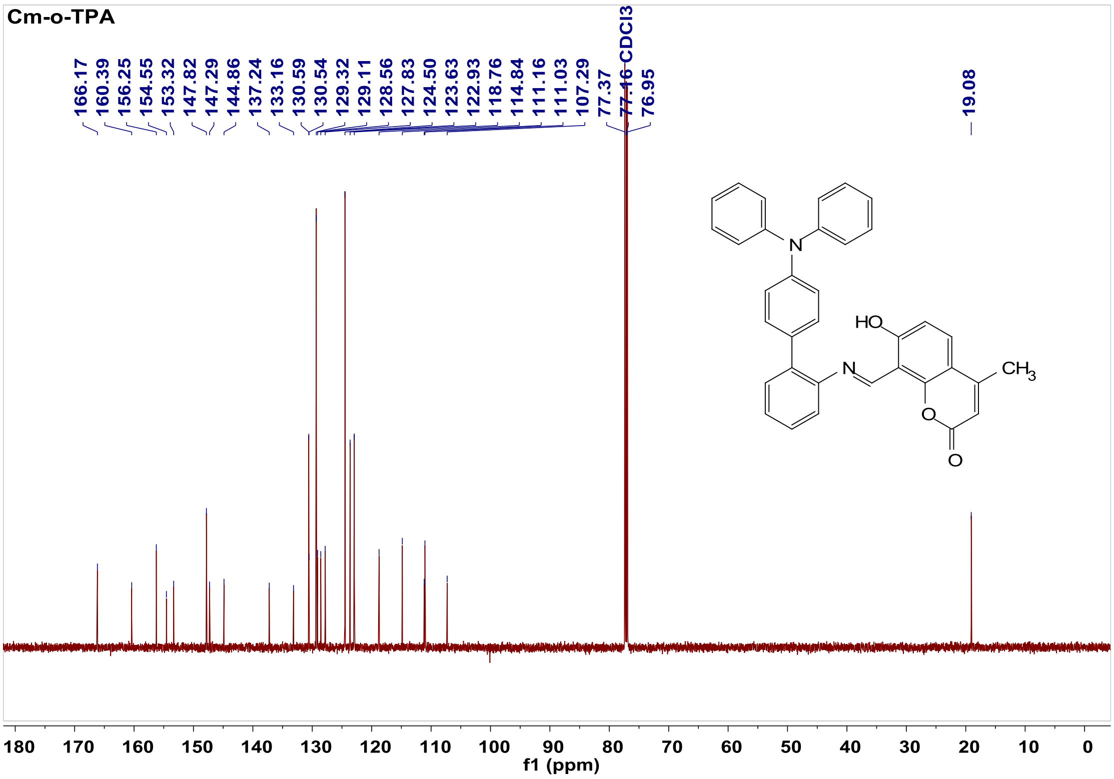

Figure S3. ^{13}C NMR spectrum of Cm-*o*-TPA.

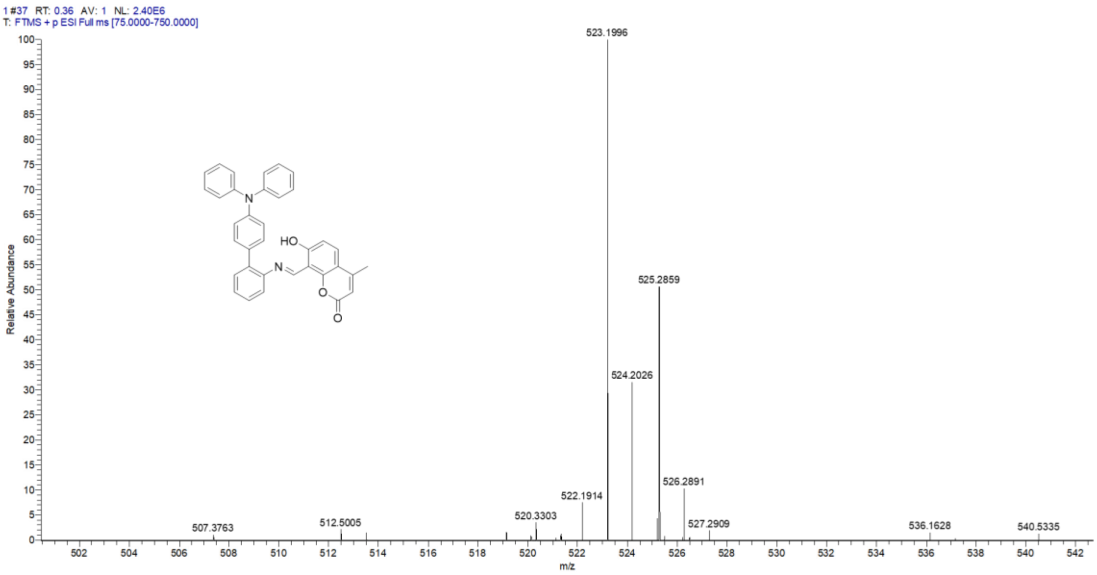

Figure S4. HR-MS spectrum of Cm-*o*-TPA.

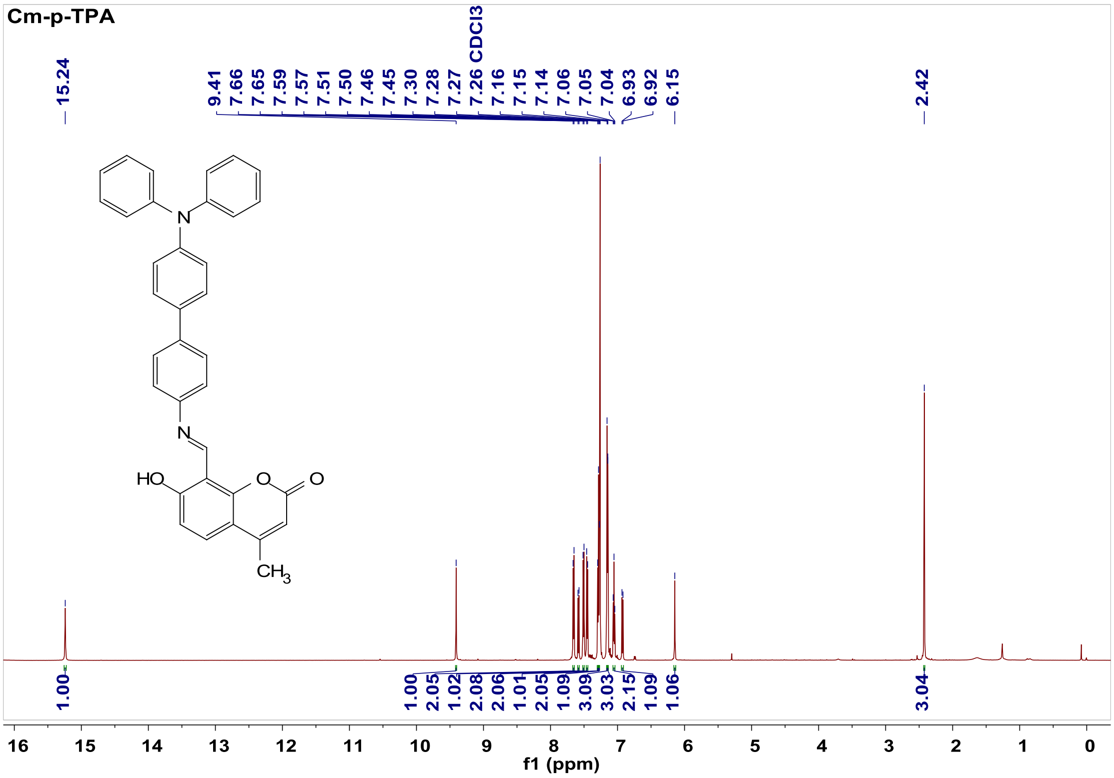

Figure S5. ^{1}H NMR spectrum of Cm-*p*-TPA.

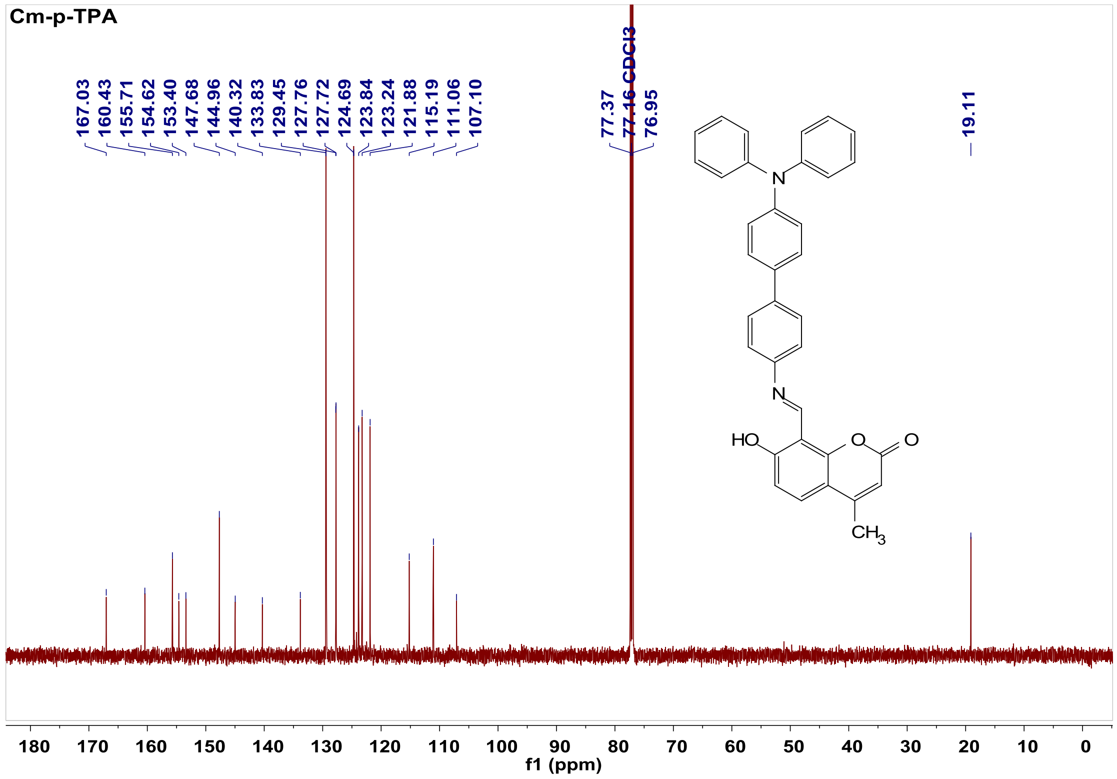

Figure S6. ^{13}C NMR spectrum of Cm-*p*-TPA.

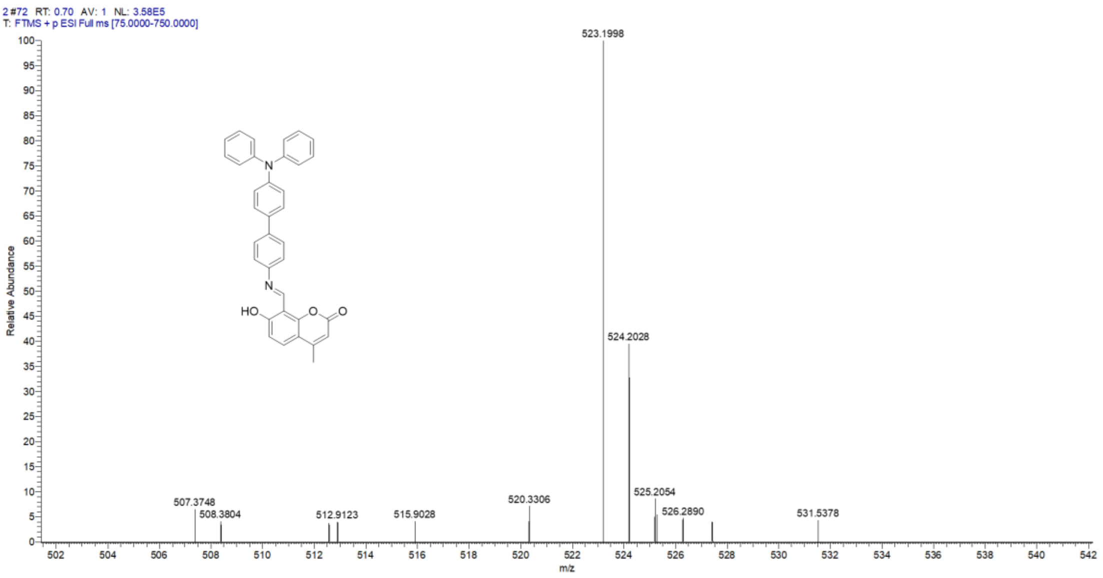

Figure S7. HR-MS spectrum of Cm-*p*-TPA.

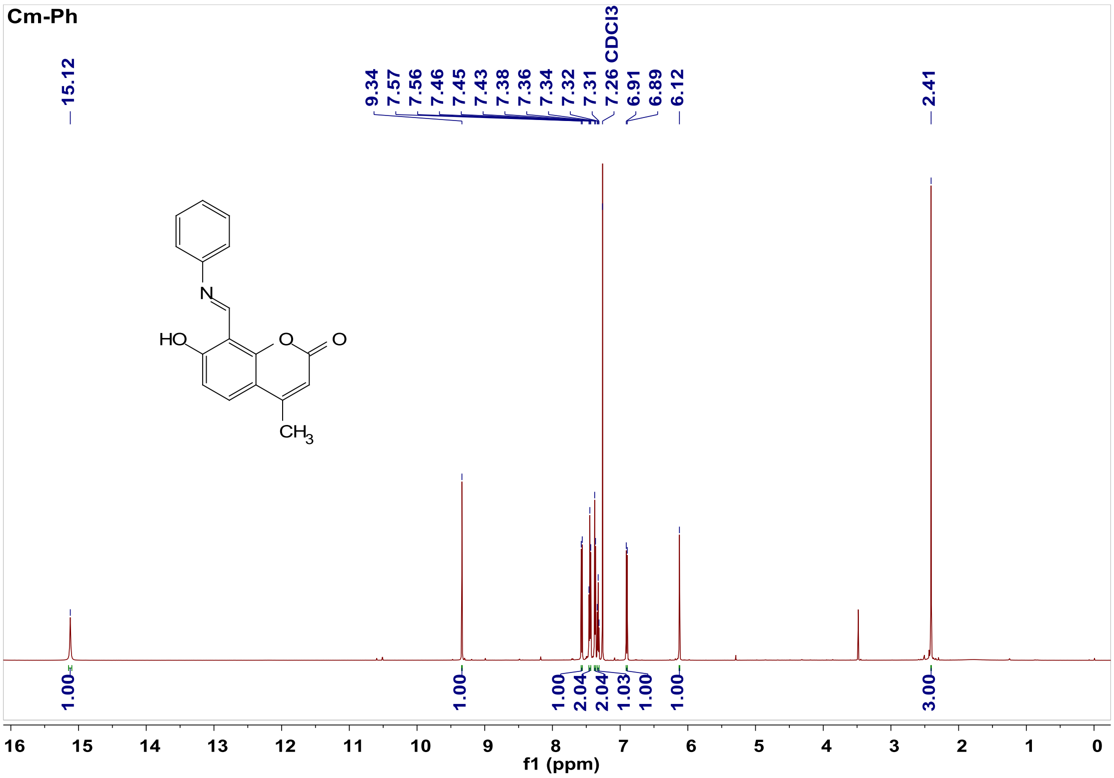

Figure S8. ^{1}H NMR spectrum of Cm-Ph.

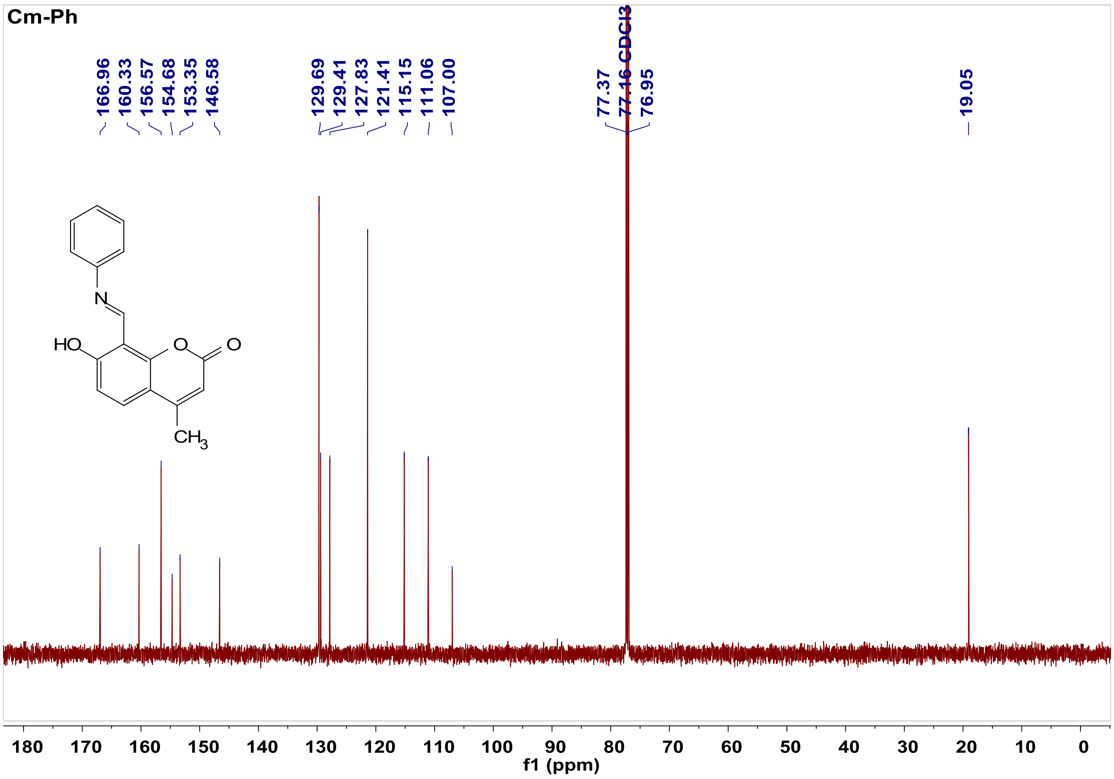

Figure S9. ^{13}C NMR spectrum of Cm-Ph.

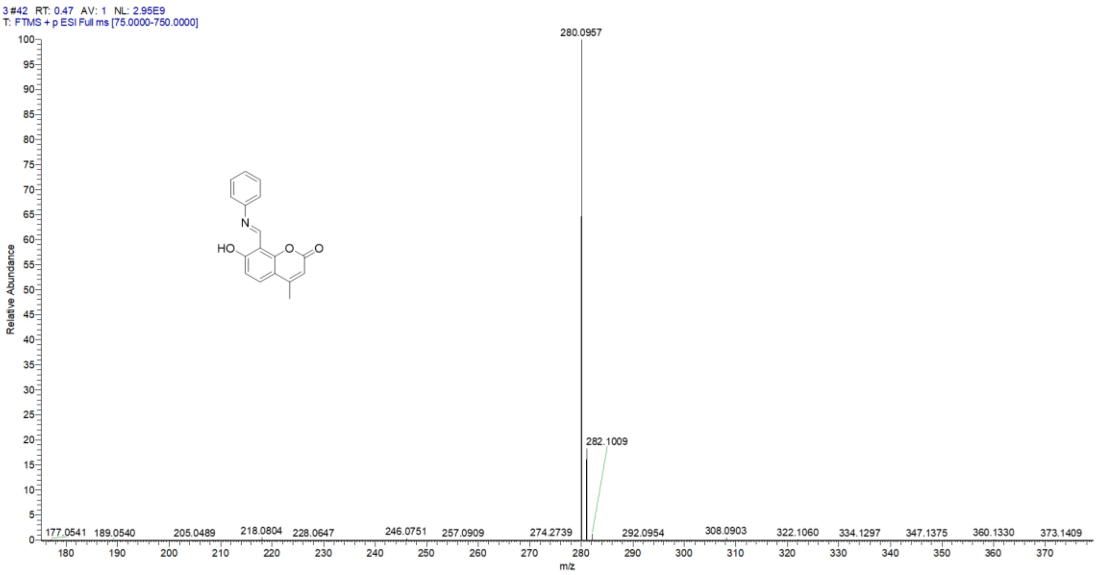

Figure S10. HR-MS spectrum of Cm-Ph.

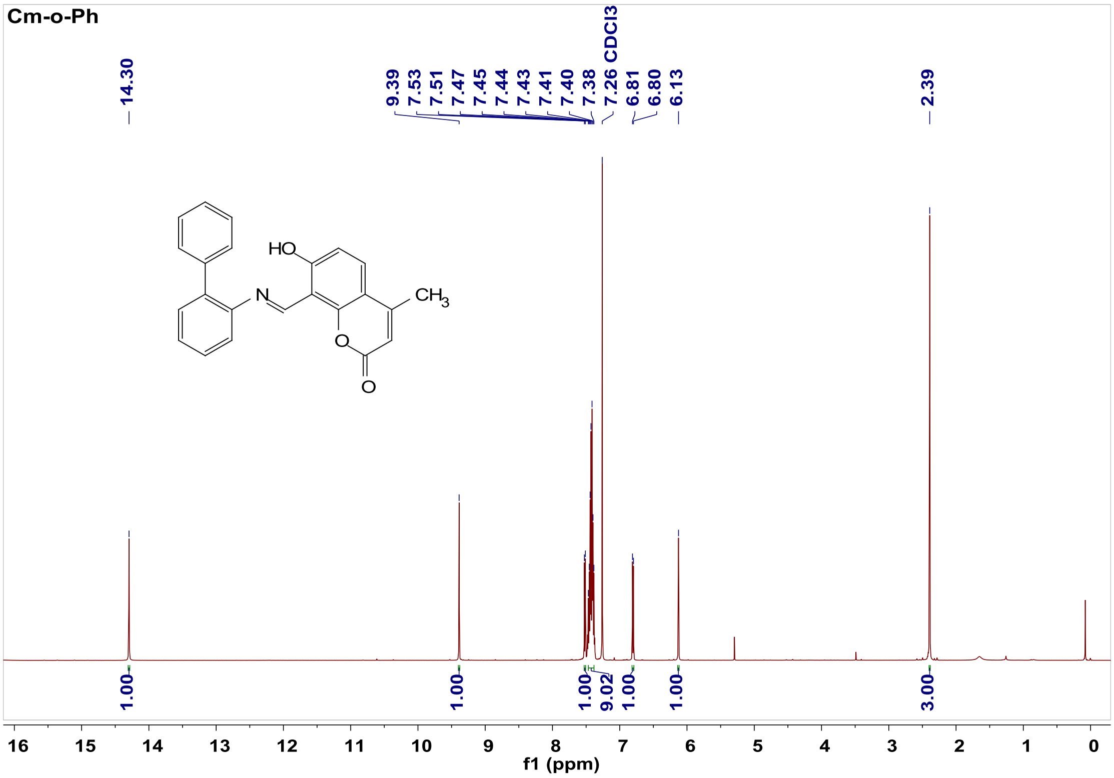

Figure S11. ^{1}H NMR spectrum of Cm-*o*-Ph.

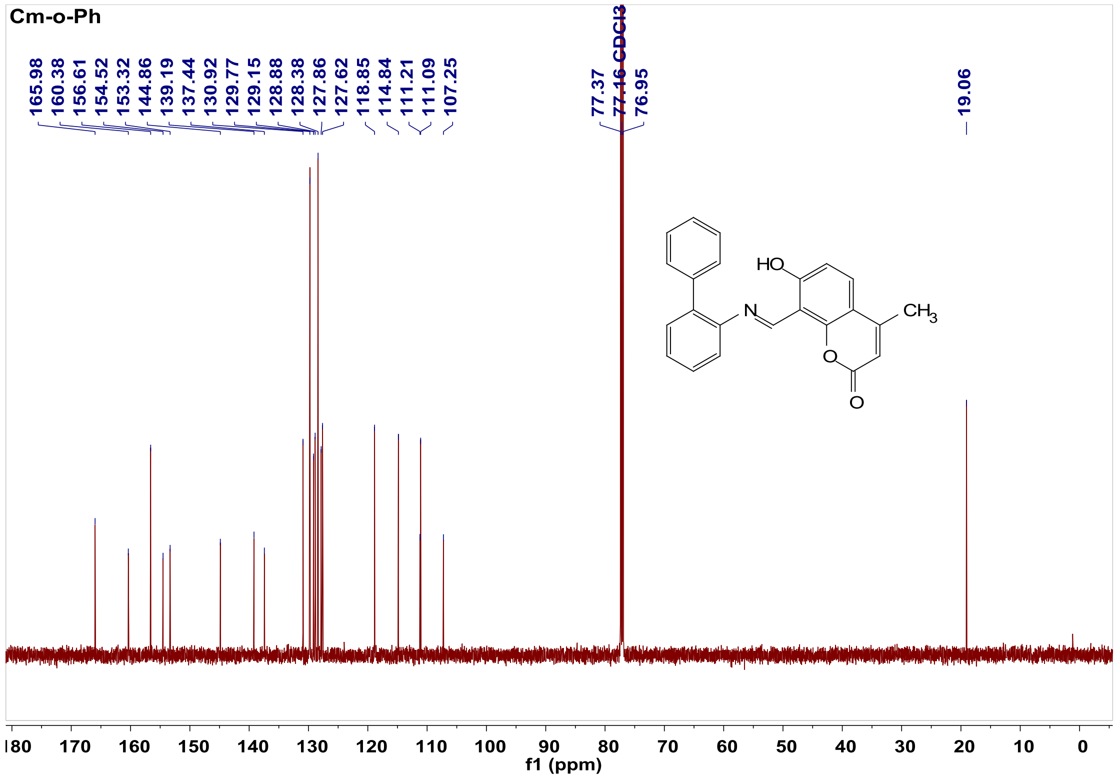

Figure S12. ^{13}C NMR spectrum of Cm-*o*-Ph.

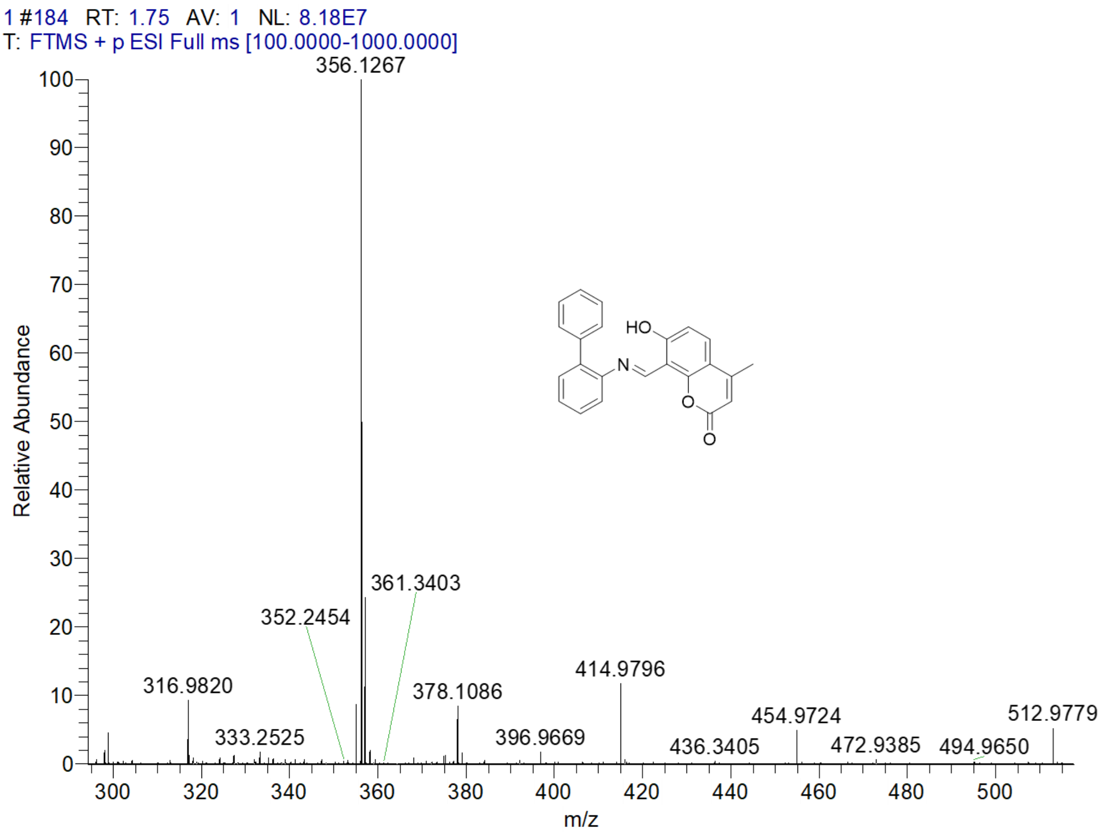

Figure S13. HR-MS spectrum of Cm-*o*-Ph.

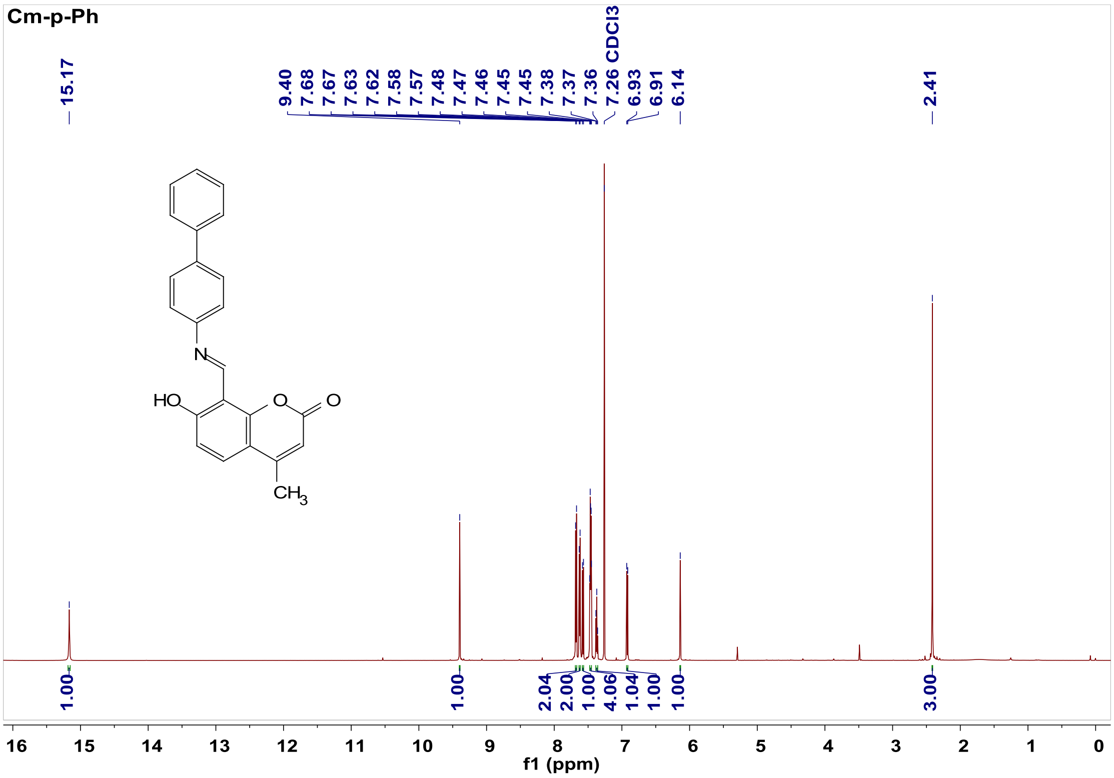

Figure S14. ^{1}H NMR spectrum of Cm-*p*-Ph.

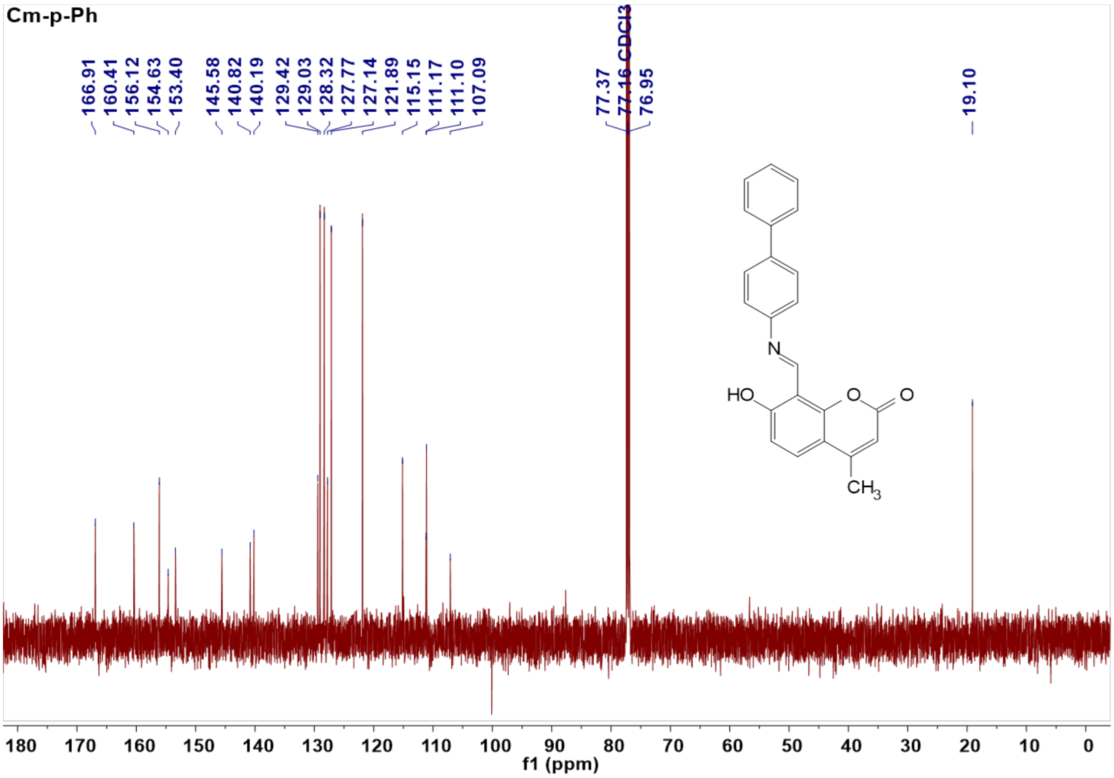

Figure S15. ^{13}C NMR spectrum of Cm-*p*-Ph**.**

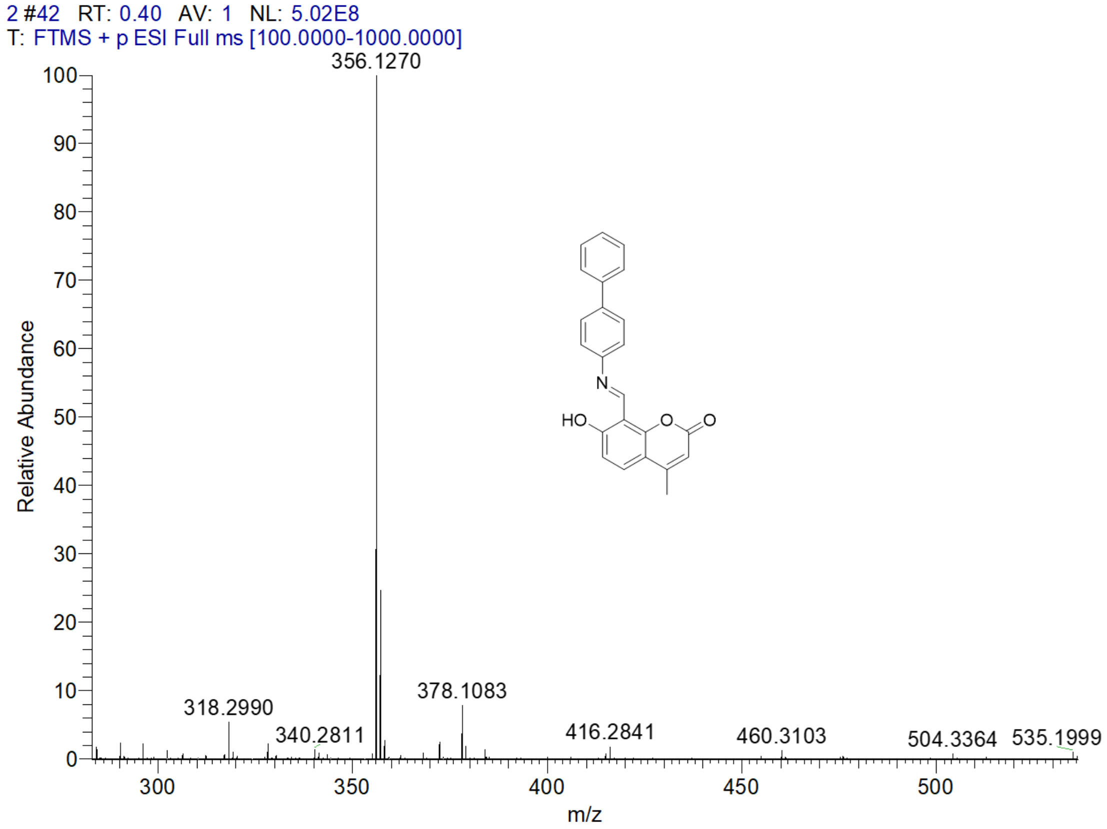

Figure S16. HR-MS spectrum of Cm-*p*-Ph.

Table S1. The QY data of Cm-*o*-TPA (λ_{ex}: 360 nm) and Cm-*p*-TPA (λ_{ex}: 380 nm) in THF/H_2O mixtures with f_w = 0%, 90%, and as solid. Inset: fluorescence photographs of Cm-*o*-TPA and Cm-*p*-TPA in THF/H_2O mixtures with f_w = 0%, 90%, and as solid taken under 365 nm UV irradiation.

QY (%) / Compd.	f_w = 0%		f_w = 90%		Solid	
Cm-*o*-TPA	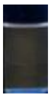	3.10	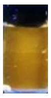	9.52	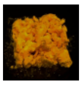	12.70
Cm-*p*-TPA	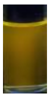	5.72	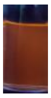	1.09	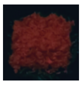	0.60

Table S2. Crystallographic data for compound Cm-*p*-TPA.

	**Cm-*p*-TPA**
empirical formula	$C_{35}H_{26}N_2O_3$
M_r	522.58
cryst syst	triclinic
space group	*P*-1
a (Å)	9.259(2)
b (Å)	9.9024(16)
c (Å)	15.160(3)
α (º)	95.768(14)
β (º)	103.28(2)
γ (º)	93.053(17)
V (Å^3)	1341.6(5)
Z	2
ρ_c (g cm^{-3})	1.294
F (000)	548.0
T (K)	293(2)
μ (mm^{-1})	0.659
data / restraints / parameters	4088/1/363
GOF (F^2)	0.837
R_1[a], wR_2[b] ($I > 2\sigma(I)$)	0.1147, 0.2328
R_{int}	0.1926

[a]$R_1 = \sum(||F_o| - |F_c||)/\sum|F_o|$; [b]$wR_2 = \{\sum[w(F_o^2 - F_c^2)^2] / \sum[w(F_o^2)^2]\}^{1/2}$

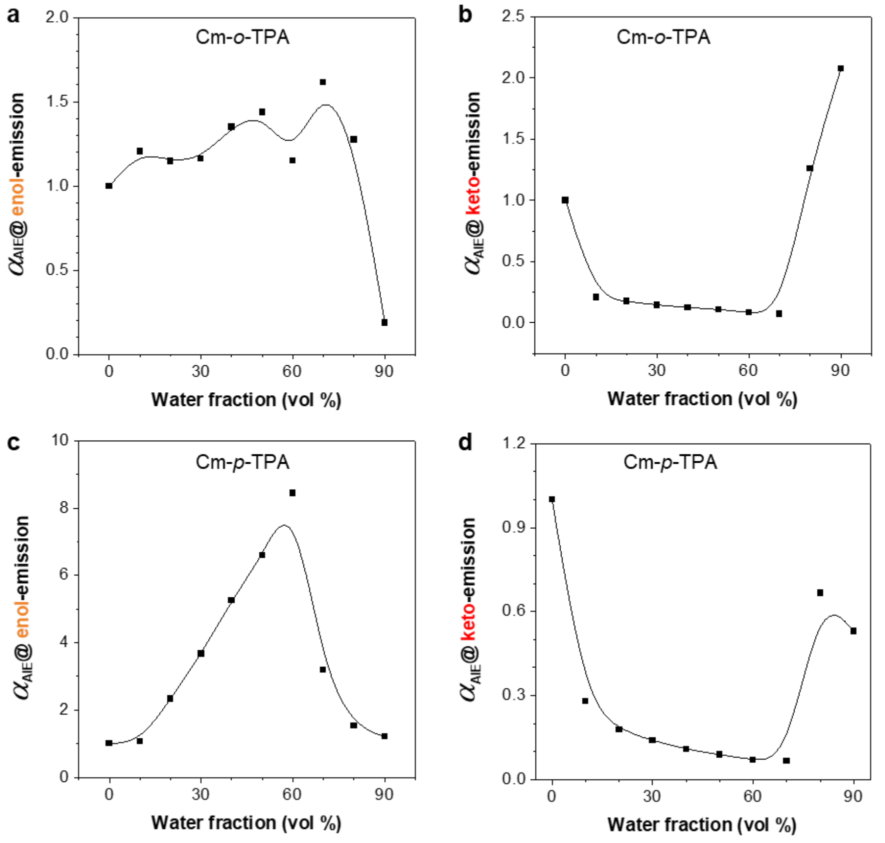

Figure S17. (a and c) The enol (α_{AIE}) emission plots (versus f_w) of Cm-*o*-TPA (a) and Cm-*p*-TPA (c). α_{AIE} = I/I_0, I_0 = PL intensity in pure THF. (b and d) The plots of the keto (α_{AIE}) emission plots (versus f_w) of Cm-*o*-TPA (b) and Cm-*p*-TPA (d). α_{AIE} = I/I_0, I_0 = PL intensity in pure THF.

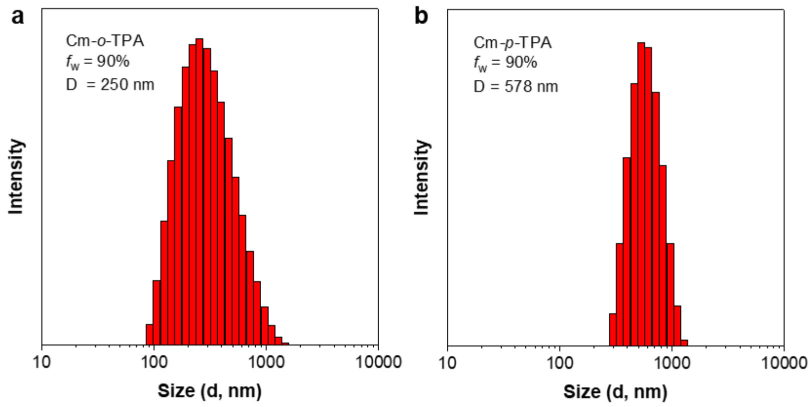

Figure S18. DLS results of Cm-*o*-TPA (a) and Cm-*p*-TPA (b) in THF/H_2O mixtures with f_w = 90% (10 μM).

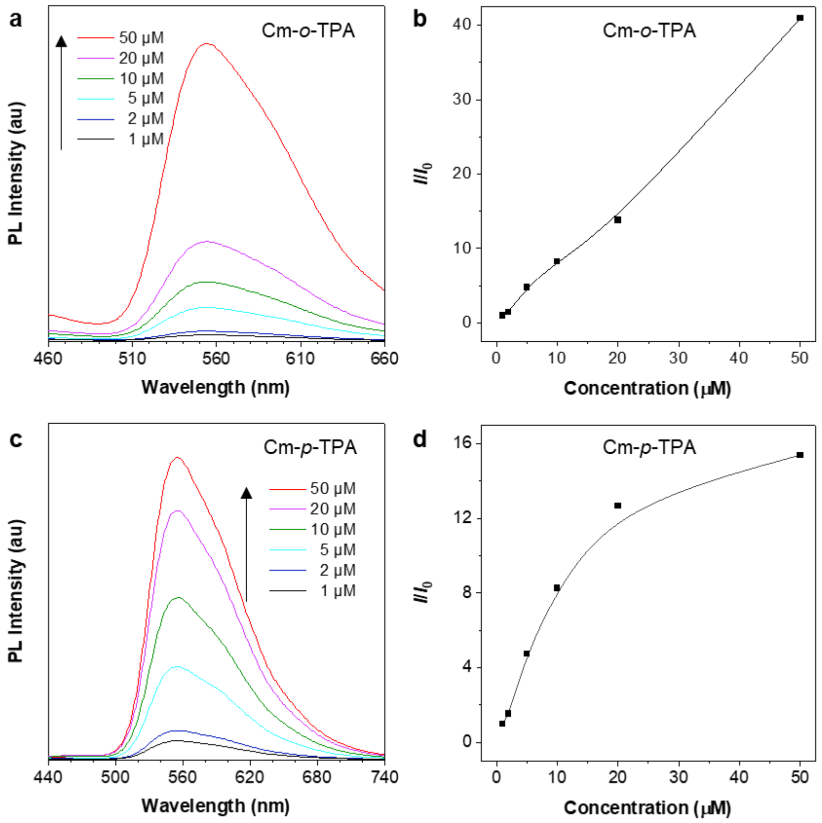

Figure S19. (a and c) PL spectra of Cm-*o*-TPA (a) and Cm-*p*-TPA (c) in THF solution with different concentrations. Cm-*o*-TPA (λ_{ex}: 360 nm) and Cm-*p*-TPA (λ_{ex}: 380 nm). (b and d) The plots of the emission intensity at the maximum versus the concentration of Cm-*o*-TPA (b) and Cm-*p*-TPA (d) in THF, I_0 = PL intensity in THF solution (1 μM).

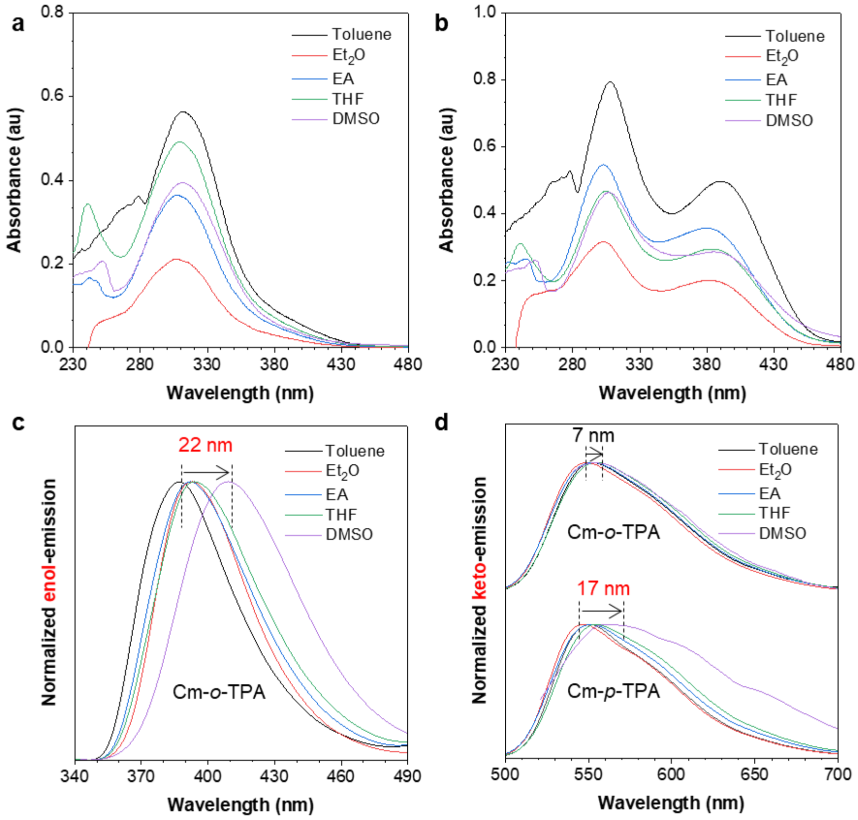

Figure S20. (a and b) Absorption spectra of Cm-*o*-TPA (a) and Cm-*p*-TPA (b) in solvents with different polarities. Concentration: 10 μM. (c) Normalized enol-emission PL spectra of Cm-*o*-TPA in solvents with different polarities. (d) Normalized keto-emission PL spectra of Cm-*o*-TPA and Cm-*p*-TPA in solvents with different polarities.

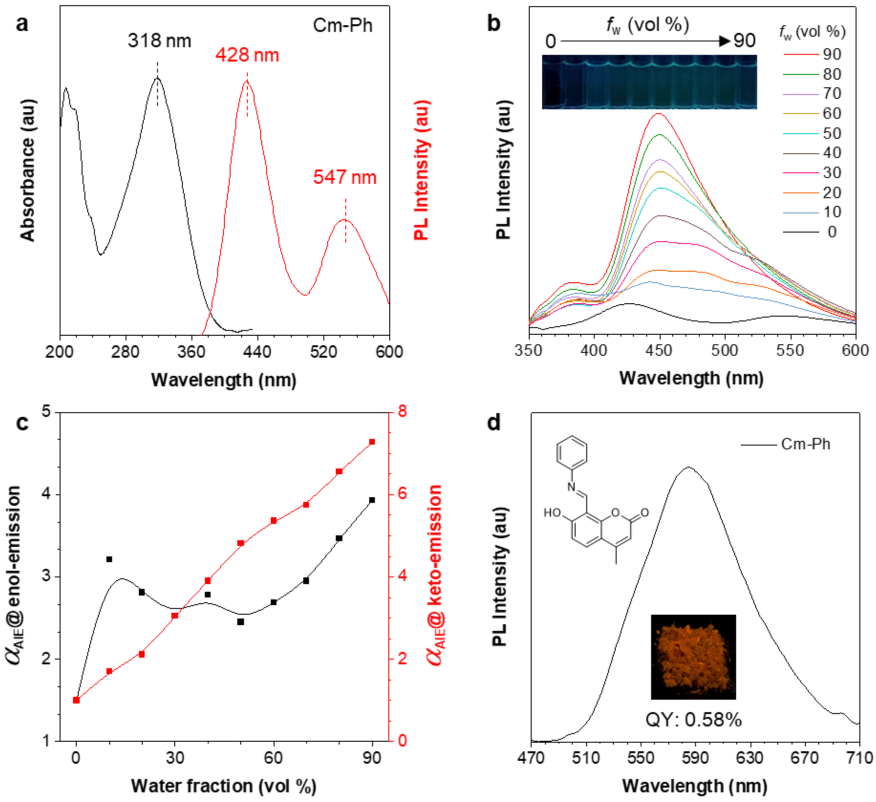

Figure S21. (a) Absorption and PL spectra of Cm-Ph in dilute ACN solution (10 μM). (λ_{ex}: 365 nm). (b) PL spectra of Cm-Ph in ACN/H_2O mixtures with different water fractions (f_w). Concentration: 10 μM. (λ_{ex}: 365 nm). Inset: The fluorescence photographs of Cm-Ph in ACN/H_2O mixtures with different water fractions (f_w). (c) The enol, keto (α_{AIE}) emission plots (versus f_w) of Cm-Ph. α_{AIE} = I/I_0, I_0 = PL intensity in pure ACN. (d) PL spectra of Cm-Ph as solid taken under 365 nm UV light. Inset: Fluorescence photographs Cm-Ph in solid states taken under 365 nm UV irradiation.

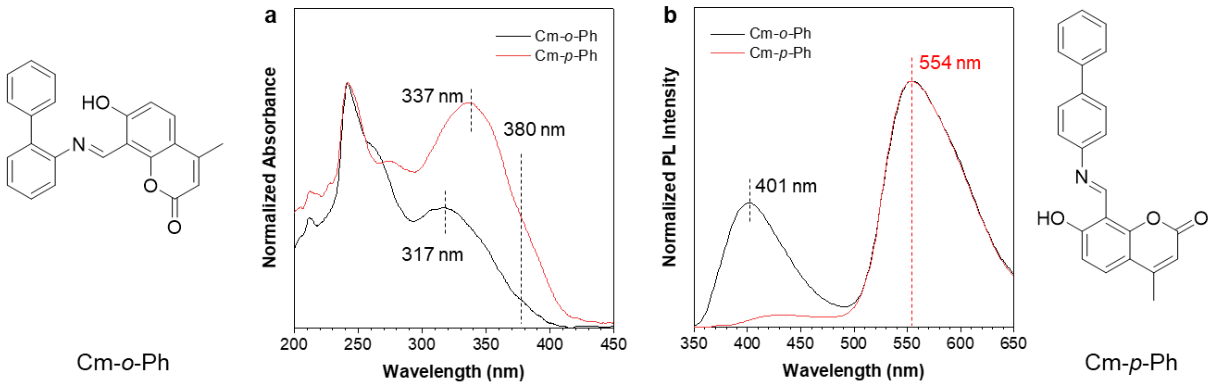

Figure S22. (a and b) Normalized absorption (a) and PL (b) spectra of Cm-*o*-Ph and Cm-*p*-Ph in pure THF solution. Concentration: 10 μM. Cm-*o*-Ph (λ_{ex}: 317 nm) and Cm-*p*-Ph (λ_{ex}: 337 nm). Insert: chemical structure of Cm-*o*-Ph and Cm-*p*-Ph.

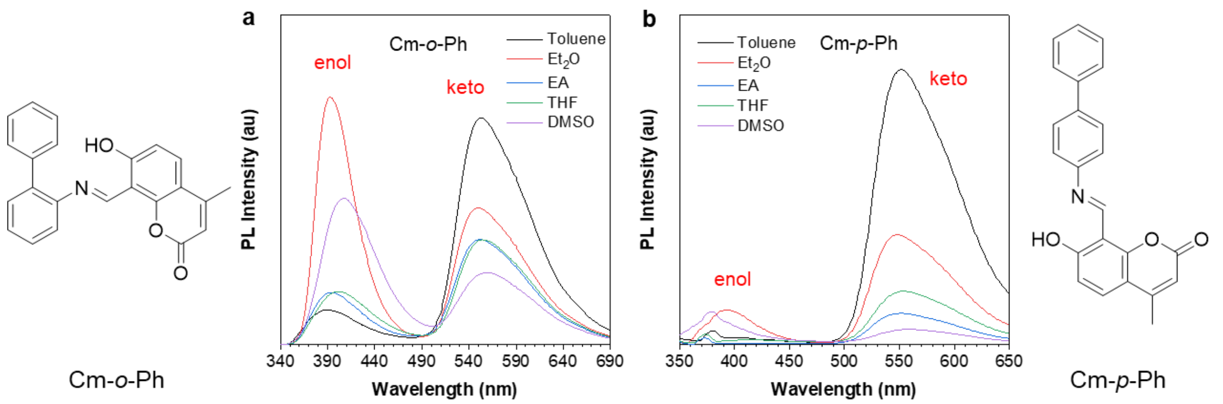

Figure S23. (a and b) PL spectra of (a) Cm-*o*-Ph and (b) Cm-*p*-Ph in solvents with different polarities. Concentration: 10 μM. The absorption maximum of each solution was chosen as its excitation wavelength.

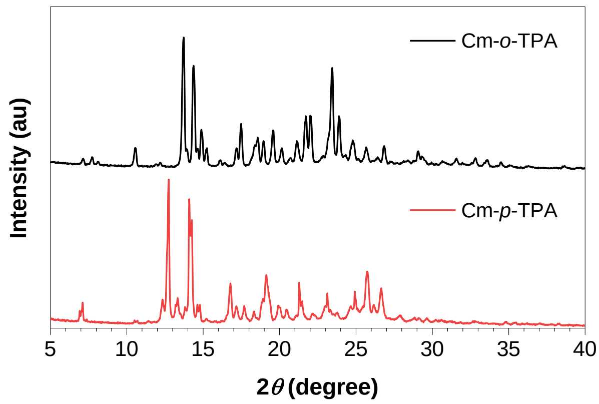

Figure S24. PXRD spectra of Cm-*o*-TPA and Cm-*p*-TPA.

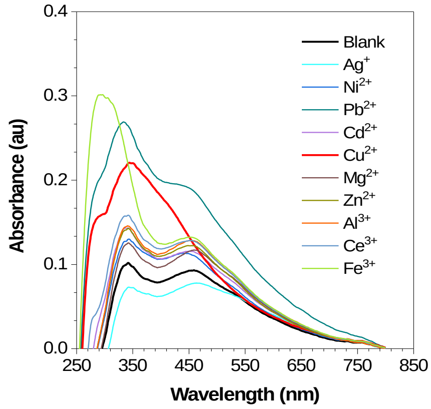

Figure S25. The absorption spectra of Cm-*p*-TPA (10 μM) in the presence of different metal ions (100 μM) including Cu^{2+} in THF/PBS (v/v = 20/80, pH = 7.4).

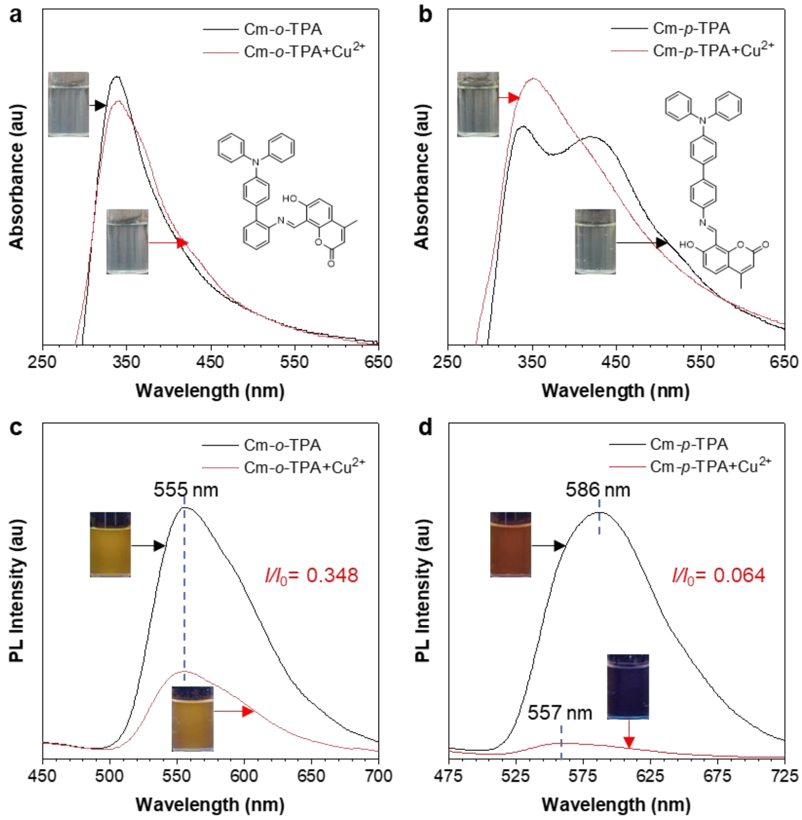

Figure S26. (a and b) Absorption spectra before and after adding Cu^{2+} (100 μM) to Cm-*o*-TPA (10 μM) (a) and Cm-*p*-TPA (10 μM) (b) in THF/PBS (v/v = 20/80, pH = 7.4) solution. Inset: the photography before and after adding Cu^{2+} to Cm-*o*-TPA and Cm-*p*-TPA in THF/PBS (v/v = 20/80, pH = 7.4) solution under day light. (c and d) PL spectra before and after adding Cu^{2+} (100 μM) to Cm-*o*-TPA (10 μM) (c) and Cm-*p*-TPA (10 μM) (d) in THF/PBS (v/v = 20/80, pH = 7.4) solution, Cm-*o*-TPA, λ_{ex} = 360 nm; Cm-*p*-TPA, λ_{ex} = 380 nm. I_0 = PL intensity before adding Cu^{2+}. I = PL intensity after adding Cu^{2+}. Inset: the photography before and after adding Cu^{2+} to Cm-*o*-TPA and Cm-*p*-TPA in THF/PBS (v/v = 20/80, pH = 7.4) solution under 365 nm UV lamp.

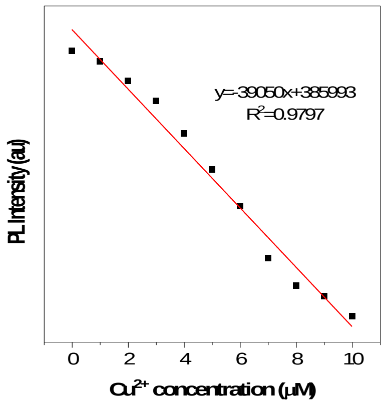

Figure S27. The linear relationships between the fluorescence intensity of Cm-*p*-TPA at 594 nm and Cu^{2+} concentration.

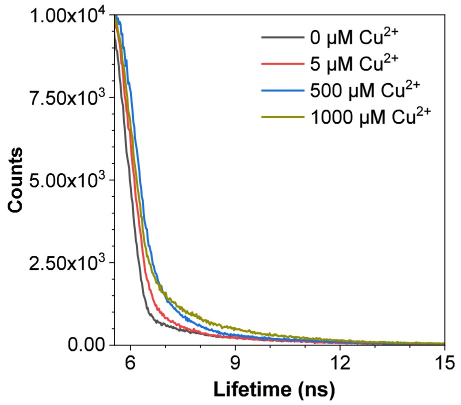

Figure S28. The lifetime decay curves of Cm-*p*-TPA with increasing amount of Cu^{2+} in PBS buffer (pH = 7.4).

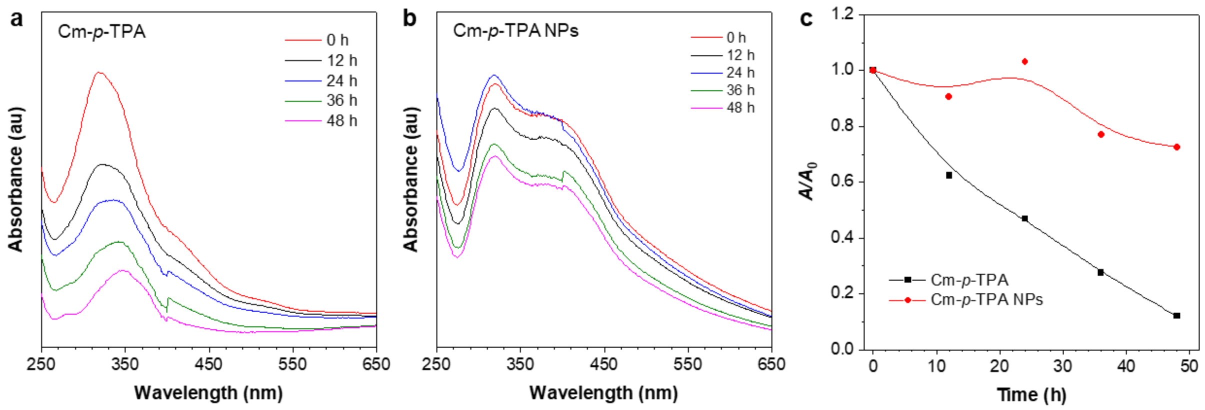

Figure S29. (a and b) Absorption spectra of Cm-*p*-TPA (a) and Cm-*p*-TPA NPs (b) in biological medium including FBS and DMEM for 48 h. (c) The plots of the absorption intensity at the maximum versus time. A_0 = Absorption intensity at 0 h. Concentration: 10 μM.

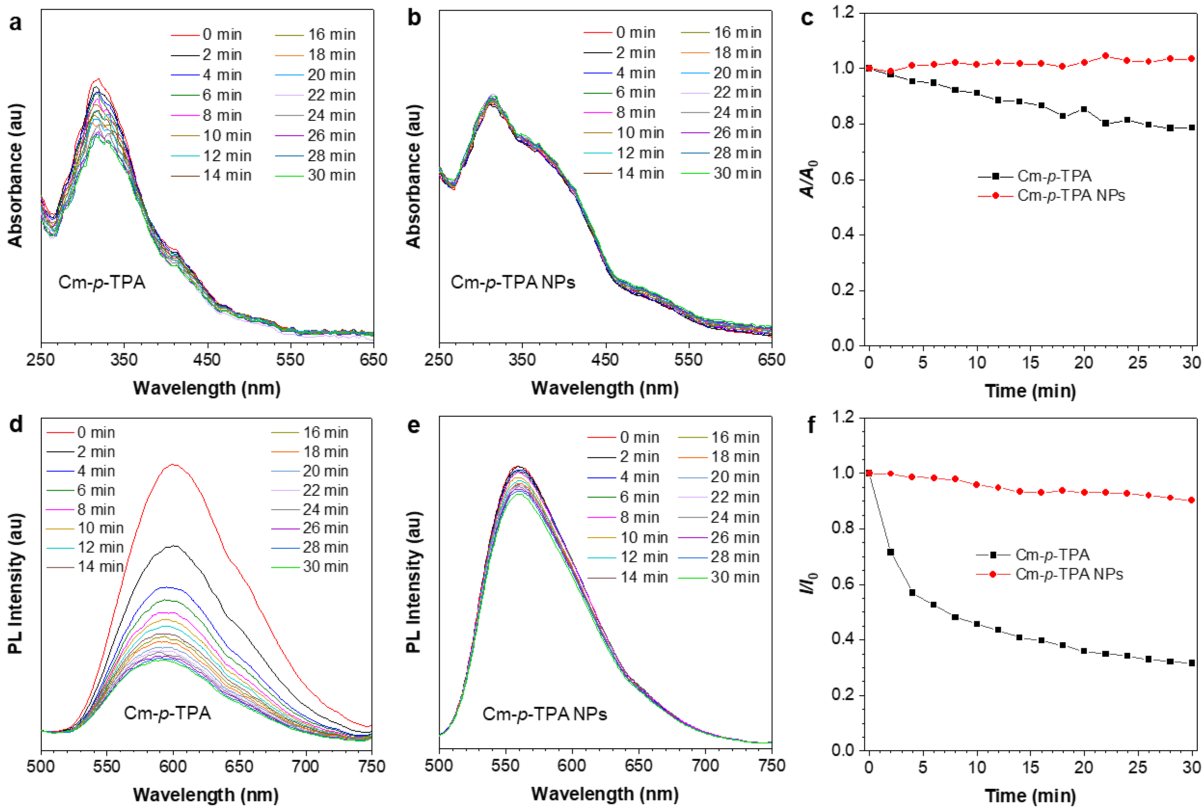

Figure S30. Absorption and PL spectra of Cm-*p*-TPA (a and d) andCm-*p*-TPA NPs (b and e) in biological medium including FBS and DMEM under white light exposure for a duration of 30 minutes. (c and f) The plots of the absorption intensity (c) and PL intensity (f) at the maximum versus time under white light exposure. A_0 = Absorption intensity at 0 min. I_0 = PL intensity at 0 min. Concentration: 10 µM.

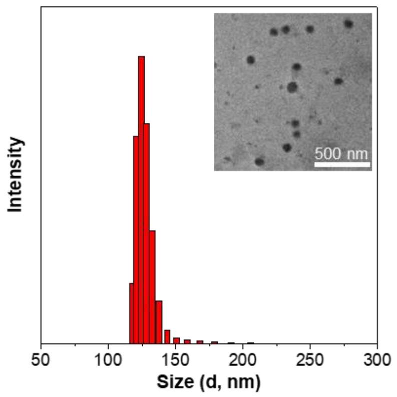

Figure S31. Hydrodynamic diameter of the Cm-*p*-TPA NPs and their distribution measured by DLS. The insert is the TEM image of theCm-*p*-TPA NPs. Scale bar: 500 nm.

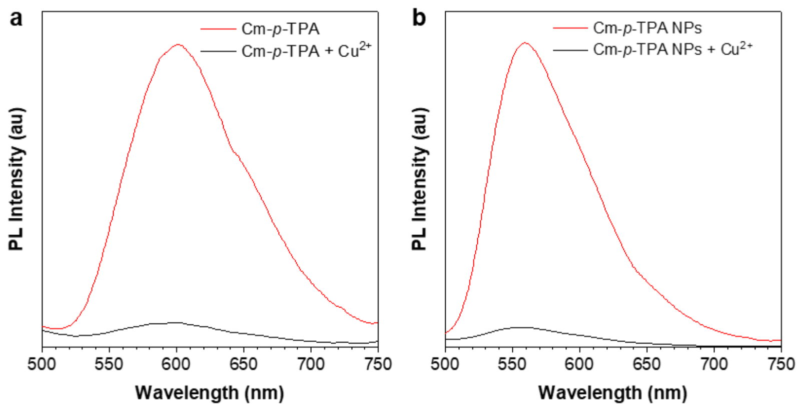

Figure S32. PL spectra before and after adding Cu^{2+} (100 µM) to Cm-*p*-TPA (10 µM) (a) and Cm-*p*-TPA NPs (b) in biological medium including FBS and DMEM.

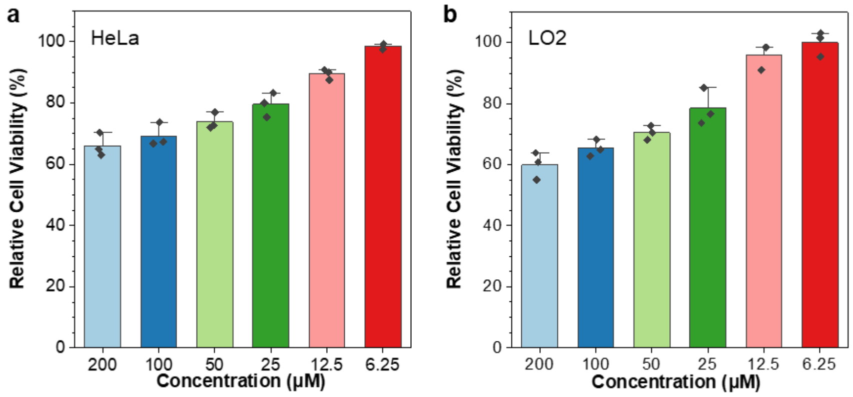

Figure S33. (a and b) Cell viabilities of HeLa (a) and LO2 (b) cells after treatment with indicated concentration of Cm-*p*-TPA NPs for 24 h. The control groups were treated with the same volume of PBS buffer without Cm-*p*-TPA NPs.

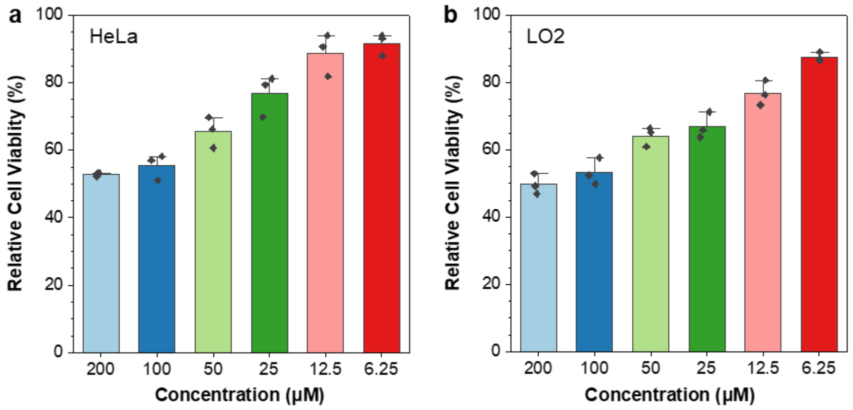

Figure S34. (a and b) Cell viabilities of HeLa (a) and LO2 (b) cells after treatment with the indicated concentration of Cm-*p*-TPA NPs for 12 h followed by irradiation using a 405 nm

led array (10 mW cm^{-2}, 10 min). Then the cells were incubated for another 12 h. The control groups were treated with the same volume of PBS buffer without Cm-*p*-TPA NPs.

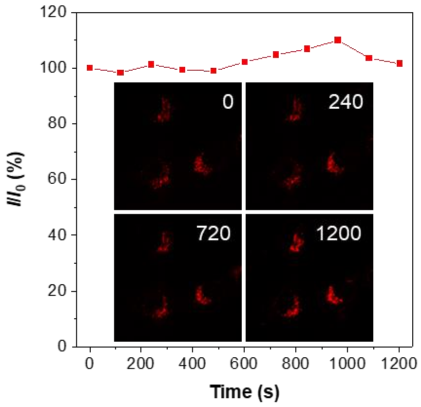

Figure S35. Photostability of Cm-*p*-TPA NPs (10 μM) in HeLa cells under continuous one-photon laser irradiation.λ_{ex} = 405 nm; λ_{em} = 590 ± 20 nm.

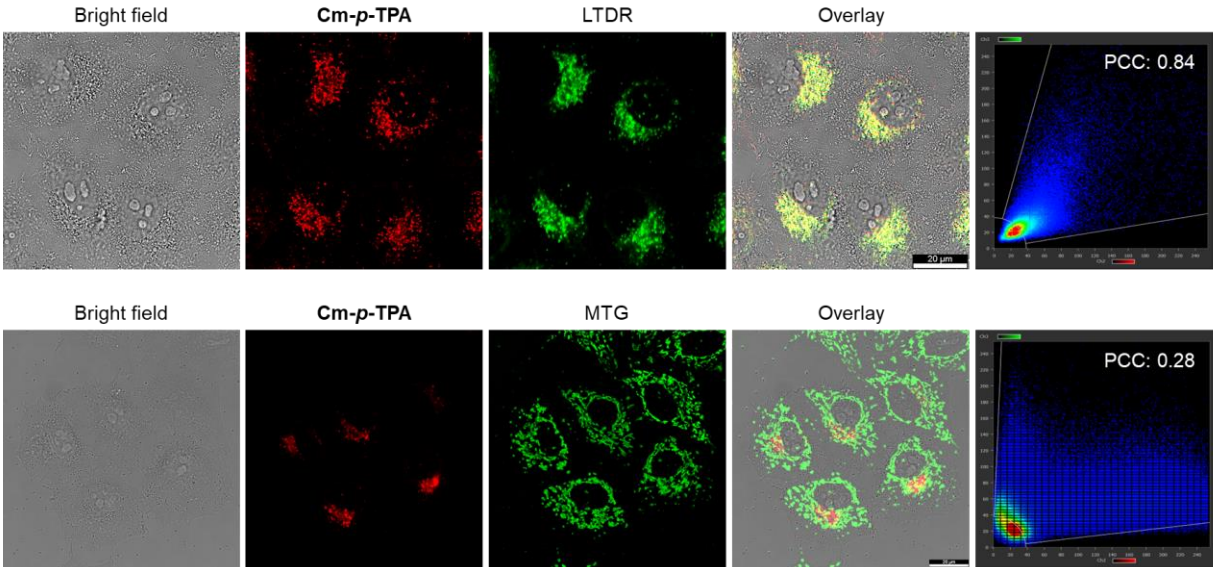

Figure S36. Co-Localization of Cm-*p*-TPA NPs (10 μM, 2 h) in HeLa cells coincubated with LysoTracker® Deep Red FM (LTDR, 200 nM, 15 min) and MitoTracker® Green FM (MTG, 200 nM, 15min). Cm-*p*-TPA NPs: λ_{ex} = 405 nm; λ_{em} = 590 ± 20 nm. LTDR: λ_{ex} = 633 nm; λ_{em} = 670 ± 20 nm. MTG: λ_{ex} = 490 nm; λ_{em} = 516 ± 20 nm. Scale bars: 20.0 μm.

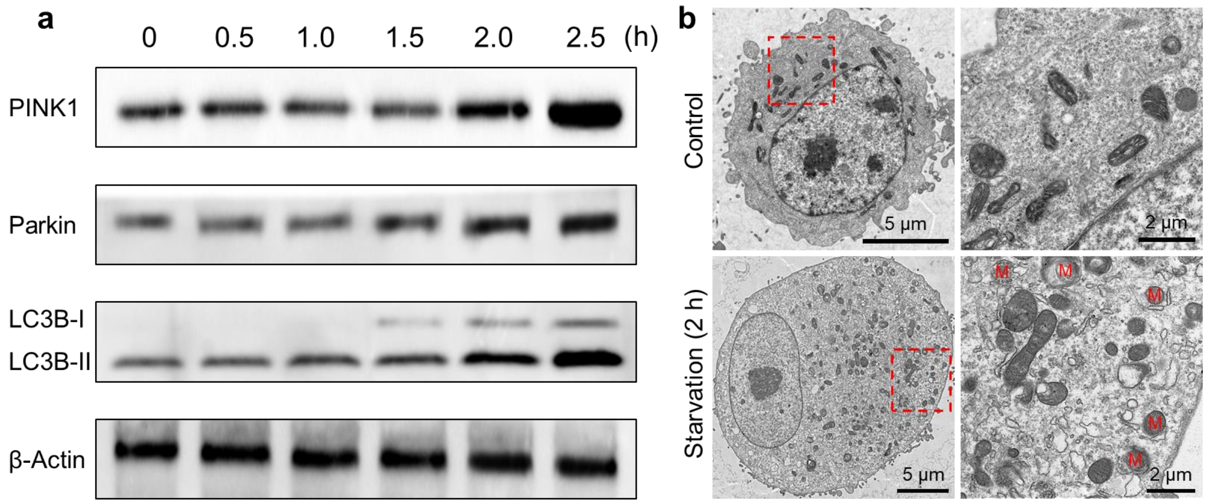

Figure S37. (a) Western blot analysis of starvation inducing mitophagy. PINK1, PTEN induced putative kinase 1. LC3, microtubule-associated protein 1 light chain 3. (b) Representative TEM images showing the ultrastructure of HeLa cells mitophagy brings after incubating in D-Hank buffer for 2 h. The cells in control groups are incubated in DMEM with 10% FBS as supplements. The red rectangle indicates the enlarged region. M represents for mitophagy.

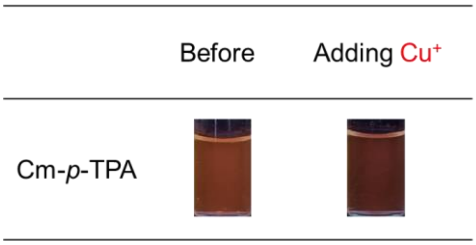

Figure S38. The photography of Cm-*p*-TPA (10 μM) before and after adding Cu^+ in THF/H_2O (v/v = 20/80) under day light and 365 nm UV lamp.

References

[1] F. Luan, G. Xiao, Y. Zhang, S. Li, Z. Hu, H. Du and D. Guo, *J. Mol. Liq.* **2020**, *320*, 114439.

Author Contributions

Xu-Min Cai: Conceptualization, Methodology, Formal analysis, Resources, Writing-Review & Editing, Visualization, Supervision, Project administration, Funding acquisition. **Shouji Li**: Validation, Formal analysis, Investigation, Writing-Original Draft. **Wen-Jin Wang**: Validation, Formal analysis, Investigation, Writing-Review & Editing. **Yuting Lin**: Formal analysis, Investigation. **Weiren Zhong**: Formal analysis, Investigation. **Yalan Yang**: Investigation. **Fritz E. Kühn**: Writing-Review & Editing. **Ying Li**: Investigation. **Zheng Zhao**: Supervision. **Ben Zhong Tang**: Supervision, Funding acquisition.
